# Supplementary material for: Socioeconomic trajectories of body mass index and waist circumference: results from the English Longitudinal Study of Ageing
Source: BMJ Open. 2019 Apr 20;9(4):e025309. doi: 10.1136/bmjopen-2018-025309 (PMC6500398; doi:10.1136/bmjopen-2018-025309)
Supplement: Supplementary data [file bmjopen-2018-025309supp001.pdf]

Supplementary Materials

Socioeconomic status and trajectories of body mass index and waist circumference in older adults

Definition of Total non-pension wealth

During the face-to-face interview ELSA collects information on the financial circumstances of respondents, the questions are designed by the Institute for Fiscal Studies (IFS). From the 45 single components of income and 31 single components of wealth, an aggregate measure of wealth is derived by economists at IFS.

Total non-pension wealth: Total non-pension wealth is reported at the family level and is defined as the sum of net financial wealth, net physical wealth and net housing wealth.

Family: is a couple or a single person and any children aged below 18 they may have who are living at home.

Net financial wealth is reported at the family level and is defined as savings (interest-bearing current and deposit accounts, cash ISAs) plus investments (premium bonds, National Savings, PEPs, shares, trusts, bonds, the saving element of life insurance, shares ISAs and life insurance ISAs) and minus debt (outstanding balances on credit cards, loans, mail-order and other private debt but not including mortgages).

Net housing wealth: Net housing wealth is reported at the family level and is defined as the self-reported current value of primary housing (i.e. residential housing).

Net physical wealth: Net physical wealth is reported at the family level and is defined as wealth held in second homes, farm or business property, other business wealth, other land and other assets, such as jewellery, works of art or antiques.

Table S1 Sample size and attrition rates by wave of assessment

**BMI sample**	**Wave 2 (2004-2005)**	**Wave 4 (2008-2009)**	**Wave 6 (2012-2013)**
Interviewed	7,225	5,483	4,724
Dropped out		1,742	2,501
% Attrition		24.1%	34.6%
**Waist circumference sample**	**Wave 2 (2004-2005)**	**Wave 4 (2008-2009)**	**Wave 6 (2012-2013)**
Interviewed	7,416	5,609	4,821
Dropped out		1,807	2,595
Attrition		24.4%	35.0%

Table S2. Joint Model of Attrition and Latent Growth Model of BMI with age, England 2004-5 to 2012-13

	Estimate	95% CI	p-value
**Growth parameters**			
Intercept	28.17	28.03; 28.32	<0.001
Intercept variance	22.95	22.66; 23.44	<0.001
Slope	0.031	0.018; 0.044	<0.001
Slope variance	0.079	0.047; 0.112	<0.001
**Time-invariant predictors**			
Intercept regressed on			
Baseline age[a]	-0.362	-0.474;-0.250	<0.001
Quadratic effect of age[a]	-0.274	-0.370;-0.178	<0.001
Slope regressed on			
Baseline age[a]	-0.060	-0.070;-0.050	<0.001
Quadratic effect of age[a]	-0.018	-0.029; -0.007	0.007
**Loss to follow up at wave 4 on**	**OR**	**95% CI**	**p-value**
BMI	1.007	0.996;1.012	0.209
Baseline age[a]	1.515	1.439;1.596	<0.001
Quadratic effect of age[a]	1.228	1.172;1.288	<0.001
**Loss to follow up at wave 6 on**			
BMI	1.011	1.001;1.021	0.039
Baseline age[a]	1.842	1.754;1.934	<0.001
Quadratic effect of age[a]	1.368	1.299;1.440	<0.001
N	7,225		

[a] Baseline age is centred at the grand mean of 66 years and presented in 10years increment

Table S3 Joint Model of Attrition and Latent Growth Model of waist circumference with age, England 2004-5 to 2012-13

	Estimate	95% CI	p-value
**Growth parameters**			
Intercept	96.71	96.30,97.12	<0.001
Intercept variance	157.03	150.49,163.57	<0.001
Slope	0.196	0.159,0.234	<0.001
Slope variance	0.233	0.0.92,0.374	0.001
**Time-invariant predictors**			
Intercept regressed on			
Baseline age[a]	0.128	-0.248, 0.468	0.556
Quadratic effect of age[a]	-0.009	-0.012;-0.006	<0.001
Slope regressed on			
Baseline age[a]	-0.089	-0.120;-0.058	<0.001
Quadratic effect of age[a]	-0.037	-0.030; -0.006	0.029
**Loss to follow up at wave 4 on**	**OR**	**95% CI**	**p-value**
Waist circumference	1.007	1.003;1.011	0.001
Baseline age[a]	1.538	1.349;1.705	<0.001
Quadratic effect of age[a]	1.221	1.220;1.502	<0.001
**Loss to follow up at wave 6 on**			
Waist circumference	1.007	1.003;1.011	<0.001
Baseline age[a]	1.842	1.750;1.930	<0.001
Quadratic effect of age[a]	1.349	1.339;1.420	<0.001
N	7,416		

[a] Baseline age is centred at the grand mean of 66 years and presented in 10years increment

Table S4. P-values for interaction terms between sex and wealth in the fully adjusted Latent Growth Model of BMI and WC, ELSA 2004/2005 to 2012/2013

	BMI	WC
	**p-value**	**p-value**
**Intercept regressed on:**		
Poorest wealth & Sex Interaction	<0.010	<0.001
2nd wealth quintile & Sex Interaction	<0.010	<0.001
3^{3rd} wealth quintile & Sex Interaction	<0.010	<0.050
4th wealth quintile & Sex Interaction	<0.010	<0.050
Overall significance	<0.010	<0.010
**Slope regressed on:**		
Poorest wealth & Sex Interaction	0.081	0.039
2nd wealth quintile & Sex Interaction	0.956	0.313
3^{3rd} wealth quintile & Sex Interaction	0.256	0.775
4th wealth quintile & Sex Interaction	0.633	0.876
Overall significance	0.184	0.152

Adjusted for marital status, and limiting long standing illness. Weighted for non-response.

Table S5. Adjusted Sex-specific Latent Growth Model of BMI by age and wealth, ELSA 2004/2005 to 2012/2013

	Men (N=3259)			Women (N=3966)		
	**Estimate[a]**	**95% CI**	**p-value**	**Estimate[a]**	**95% CI**	**p-value**
Intercept	27.50	27.24; 27.77	<0.001	27.00	26.31; 26.91	<0.001
Intercept variance	18.38	16.98; 19.79	<0.001	25.82	24.33; 27.30	<0.001
Slope	0.003	-0.019; 0.025	0.808	0.016	0.004; 0.049	0.055
Slope variance	0.094	0.057; 0.131	<0.001	0.092	0.060; 0.125	<0.001
**Intercept regressed on:**						
Baseline age[b] linear	-0.602	-0.756;-0.448	<0.001	-0.434	-0.608;-0.261	<0.001
Baseline age[b] quadratic	-0.131	-0.262;-0.001	0.098	-0.366	-0.509;-0.223	<0.001
Poorest wealth	0.920	0.487; 1.352	<0.001	2.950	2.494; 3.406	<0.001
2nd wealth quintile	0.526	0.146; 0.905	<0.050	2.029	1.630; 2.429	<0.001
33rd wealth quintile	0.703	0.337; 1.068	<0.010	2.242	1.834; 2.650	<0.001
4th wealth quintile	0.030	-0.325; 0.386	0.888	1.174	0.772; 1.575	<0.001
**Slope regressed on:**						
Baseline age[b] linear	-0.046	-0.060;-0.032	<0.001	-0.073	-0.088;-0.059	<0.001
Baseline age[b] quadratic	-0.006	-0.023; 0.010	0.526	-0.021	-0.021; -0.006	0.026
Poorest wealth	0.040	-0.001; 0.079	0.092	-0.020	-0.060; 0.019	0.402
2nd wealth quintile	0.021	-0.013; 0.054	0.308	0.022	-0.012; 0.055	0.282
33rd wealth quintile	0.046	0.014; 0.078	0.019	0.015	-0.015; 0.045	0.418
4th wealth quintile	0.012	-0.014; 0.037	0.417	0.003	-0.026; 0.031	0.884
**Model Fit**						
CFI		0.994				
TLI		0.982				
RMSEA		0.020				

CFI = Comparative Fit Index; TLI = Tucker–Lewis index; RMSEA = root-mean-square error of approximation.
[a] Estimates adjusted for marital status, and limiting long standing illness, all centered to the grand mean.
[b] Baseline age is centred at the grand mean of 66 years and presented in 10years increment
Weighted for non-response.

Table S6. Adjusted Sex-specific Latent Growth Model of Waist circumference by age and wealth, ELSA 2004/2005 to 2012/2013

	Men (N=3337)			Women (N=4079)		
	**Estimate[a]**	**95% CI**	**p-value**	**Estimate**	**95% CI**	**p-value**
Intercept	100.87	100.15; 101.58	<0.001	88.45	88.45; 101.00	<0.001
Intercept variance	114.98	107.95; 122.01	<0.001	132.77	132.27; 131.07	<0.001
Slope	0.114	0.049; 0.180	<0.010	0.231	0.091; 0.224	<0.001
Slope variance	0.281	0.130; 0.433	<0.010	0.330	0.206; 0.450	<0.001
**Intercept regressed on:**						
Baseline age[b] linear	-0.212	-0.603; -0.179	0.372	-0.382	-0.057; -0.001	0.120
Baseline age[b] quadratic	-0.474	-0.831; -0.116	<0.050	-0.922	-0.010; -0.005	<0.001
Poorest wealth	2.562	1.437; 3.688	<0.001	6.167	3.681; 5.239	<0.001
2nd wealth quintile	1.674	0.689; 2.659	<0.010	4.321	2.308; 3.705	<0.001
3^{3rd} wealth quintile	1.946	0.973; 2.920	<0.010	3.971	2.229; 3.603	<0.001
4th wealth quintile	0.297	0.627; 1.220	0.597	2.150	0.551; 1.879	<0.001
**Slope regressed on:**						
Baseline age[b] linear	-0.106	-0.145;-0.067	<0.001	-0.091	-0.131;-0.050	<0.001
Baseline age[b] quadratic	-0.061	-0.104; -0.018	0.019	-0.015	-0.060; 0.030	0.575
Poorest wealth	0.014	-0.001; 0.228	0.103	-0.090	-0.076; 0.079	0.157
2nd wealth quintile	0.095	0.000; 0.190	0.098	0.011	-0.014; 0.121	0.846
3^{3rd} wealth quintile	0.077	-0.012; 0.166	0.156	0.055	-0.008; 0.133	0.298
4th wealth quintile	-0.029	-0.113; 0.056	0.577	-0.041	-0.097; 0.026	0.045
**Model Fit**						
CFI		0.981				
TLI		0.942				
RMSEA		0.052				

CFI = Comparative Fit Index; TLI = Tucker–Lewis index; RMSEA = root-mean-square error of approximation.
[a] Estimates adjusted for marital status, and limiting long standing illness, all centered to the grand mean.
[b] Baseline age is centred at the grand mean of 66 years and presented in 10years increment.
Weighted for non-response.
