# Supplementary material for: Socioeconomic trajectories of body mass index and waist circumference: results from the English Longitudinal Study of Ageing
Source: BMJ Open. 2019 Apr 20;9(4):e025309. doi: 10.1136/bmjopen-2018-025309 (PMC6500398; doi:10.1136/bmjopen-2018-025309)
Supplement: Author's manuscript [file bmjopen-2018-025309.draft_revisions.pdf]

BMJ Open

BMJ Open is committed to open peer review. As part of this commitment we make the peer review history of every article we publish publicly available.

When an article is published we post the peer reviewers' comments and the authors' responses online. We also post the versions of the paper that were used during peer review. These are the versions that the peer review comments apply to.

The versions of the paper that follow are the versions that were submitted during the peer review process. They are not the versions of record or the final published versions. They should not be cited or distributed as the published version of this manuscript.

BMJ Open is an open access journal and the full, final, typeset and author-corrected version of record of the manuscript is available on our site with no access controls, subscription charges or pay-per-view fees (http://bmjopen.bmj.com).

If you have any questions on BMJ Open's open peer review process please email info.bmjopen@bmj.com

Socioeconomic trajectories of body mass index and waist circumference: results from the English Longitudinal Study of Ageing

Journal:	*BMJ Open*
Manuscript ID	bmjopen-2018-025309
Article Type:	Research
Date Submitted by the Author:	09-Jul-2018
Complete List of Authors:	Zaninotto, Paola; UCL, Epidemiology and Public Health<br>lassale, Camille; University College London, Epidemiology and Public Health
Keywords:	body mass index, waist circumference, Trajectories, Wealth, Ageing

SCHOLARONE™
Manuscripts

Socioeconomic trajectories of body mass index and waist circumference: results from the English Longitudinal Study of Ageing

Paola Zaninotto[1], Camille Lassale[1]

[1]Department of Epidemiology and Public Health, University College London, London, UK

Corresponding author:

Paola Zaninotto, PhD

Senior Lecturer in Medical Statistics,

Department of Epidemiology and Public Health, University College London

1-19 Torrington Place

London, WC1E 7HB, UK

p.zaninotto@ucl.ac.uk

0044(0)2076791668

Key words: Body Mass Index, Waist circumference, Trajectories, Wealth, Aging

Licence for publication: The Corresponding Author has the right to grant on behalf of all authors and does grant on behalf of all authors, an exclusive licence (or non-exclusive for government employees) on a worldwide basis to the BMJ Publishing Group Ltd to permit this article (if accepted) to be published in JECH and any other BMJPGL products and sub-licences such use and exploit all subsidiary rights, as set out in our licence (http://group.bmj.com/products/journals/instructions-for-authors/licence-forms).

For peer review only

Abstract

Objectives: To explore age-trajectories of Body Mass Index (BMI) and waist circumference (WC) and to examine whether these trajectories varied by wealth.

Design: Nationally representative prospective cohort study.

Setting: Observational study of people living in England.

Participants: 7416 participants aged 52 and over of the English Longitudinal Study of Ageing (2004-2012).

Primary outcome measures: BMI and WC assessed objectively by a trained nurse.

Results: Using latent growth curve models we showed that BMI increased by 0.03Kg/m^2 (95%CI 0.02; 0.04 p<0.001) per year and WC by 0.194cm **(**0.13,0.22, p<0.001). Age (linear and quadratic) showed a negative association with BMI and WC baseline and rates of change, indicating that older individuals had smaller body sizes and that the positive rates of change flattened to eventually become negative. The decline occurred around the age of 71y for BMI and 80y for WC. Poorest wealth was significantly related to higher baseline levels of BMI (2.2Kg/m^2 95%CI:1.78; 2.25, p<0.001) and WC (4.66cm 95%CI:3.76; 5.57, p<0.001). However, no significant difference was found in the rate of change of BMI and WC by wealth.

Conclusions: Adults aged 52 and above showed an increase in BMI and WC over time but this trend reversed at older old age to display a sharp decrease. At any given age wealthier people had more favourable BMI and WC profile.

For peer review only

Strengths and limitations of this study

- This is the first large nationally representative study in England to depict age-specific trajectories of Body Mass Index and waist circumference over an 8-year period and to examine whether these differed by wealth.
- A trained nurse assessed Body Mass Index and waist circumference objectively.
- This study employed wealth as measure of socioeconomic status that is particularly relevant at older ages, since it reflects both past and current socioeconomic status.
- The study findings are limited to men and women aged 52 and over.

For peer review only

For peer review only - http://bmjopen.bmj.com/site/about/guidelines.xhtml

Introduction

There has been a marked increase in the number of obese older adults over the past decades, and the number is set to grow as the population continues to age. In the UK it is estimated that there will be an additional 11 million obese adults by 2030, of whom 3.3 million aged over 60 years. [1] The obesity epidemic has negative health consequences also in older adults where excess adiposity has been associated with chronic diseases, [2 3] disability [4 5] and mortality. [6 7]

It is increasingly recognised that a dynamic measure of body mass index (BMI) better predicts future health risks among older adults. [8 9] In older adults it has been shown that BMI increased over time up to early old age, [10 11] and declined thereafter [12 13] due to loss in muscle mass (sarcopenia). Increasing evidence indicates differential cardiovascular risk according to adipose depots location, with increased risk observed with visceral compared to subcutaneous fat.[14]
Therefore in older adults waist circumference can be a better predictor of cardiovascular risk than BMI, and both indicators are important to monitor total and visceral adiposity.

Total and central obesity are socially patterned in developed countries with higher rates of obesity observed in lower socioeconomic groups.[15 16] However, there is limited evidence on BMI trajectories in relation to socioeconomic indicators in older adults, and results are contradictory.[10 11 17] To our knowledge, no studies from developed countries specifically reported socioeconomic trajectories of waist circumference among older adults. Furthermore, despite evidence of the importance of using wealth as an indicator of socioeconomic status of older individuals,[18-20] no studies have investigated longitudinal changes in BMI and waist circumference among older adults by wealth.

Our study has two specific aims. The first objective is to depict age-trajectories of BMI and waist circumference by analysing 8 years (2004-2005 to 2012-2013) of longitudinal data from a nationally representative sample of English adults aged 50 and over. The second objective is to examine whether these trajectories varied by wealth.

For peer review only - http://bmjopen.bmj.com/site/about/guidelines.xhtml

Methods

The data are from three waves of the English Longitudinal Study of Ageing (ELSA) ,[21] where the same individuals aged 50 and older and living in private households in England, were followed and re-interviewed every 2 years. The study began in 2002-2003 (first phase of data collection referred to as wave 1) with 11 391 individuals. For the purpose of this study, we used data from wave 2 (2004-2005), wave 4 (2008-2009) and wave 6 (2012-2013) where nurse visits took place and anthropometric measurements were taken. The sample size consisted of 7255 (5042 at wave 4 and 4256 at wave 6) for the study of BMI trajectories and 7416 (4907 at wave4 and 4147 at wave 6) for the study of waist circumference trajectories. Total follow-up time was 8 years (average 4).

Patient and public involvement statement

Patients and did not participate in the design and data analysis of this study.

Anthropometric measurements

At the three waves, data were collected using face-to-face interviews and trained nurses performed a nurse visit in the households to take measurements. Height, weight, and waist circumference (WC) were measured during the nurse visit. Height without shoes was measured using a portable stadiometer. One measurement was taken with the informants stretching to the maximum height and the head in the Frankfort plane. Weight was measured using a portable electronic scale. Informants were asked to remove their shoes and any bulky clothing. BMI was defined as weight (kg)/height (m^2) (ranged from 14.9 to 56.2 kg/m^2). WC was defined as the midpoint between the lower rib and the upper margin of the iliac crest. Two measurements were taken using a tape with an insertion buckle at one end. Circumference was recorded to the nearest even millimetre The mean values of the two valid measurements (the two that were the closest to each other, if there were three measurements) was used in the analysis (ranged between 60.8 and 108.0 cm).

Wealth

Total wealth (excluding regular pension payments, but including lump sums from private pension that have already been received but not yet consumed) was defined as financial wealth, physical wealth

For peer review only - http://bmjopen.bmj.com/site/about/guidelines.xhtml

(such as business wealth, land, or jewels), and housing wealth (primary and secondary residential housing wealth), minus debts. Total wealth was divided into five equal quintiles.

Covariates

We used a continuous variable for age (ranging from 52 to 101 at baseline). Dichotomous variables were used for sex; cohabitation status (currently living or not with a partner whether married or not); smoking status (never and ex-smoker vs current smoker); physical activity (physically active vs inactive/sedentary); and for the presence of a limiting longstanding illness.

Statistical analysis

We used latent growth curve modelling to estimate baseline status (intercept) and rate of change (linear slope) over the 8-year period. Latent factors representing intercept and slope components were derived from the three observations of anthropometric measures at wave 2 (baseline), wave 4, and wave 6. Factor loadings of the intercept component to all three observations were fixed to 1, and the linear slope component was defined by fixing the parameters to 0 (baseline, wave 2), 4 (wave 4), and 8 (wave 6) so that the slope parameter can be interpreted as the change per year. To depict age trajectories of BMI and WC we fitted models with a linear and a quadratic term of age (non-adjusted). To answer our second research question, we tested the associations of wealth tertiles with intercept and slope. The models were adjusted for age (linear and quadratic), marital status, physical inactivity, smoking status and limiting long standing illness measured at baseline. The reference category was richest wealth quintile, and age at baseline was centred at its mean (66y). To determine the fit of the models, we employed the Comparative Fit Index (CFI), the Tucker–Lewis index (TLI), and the root-mean-square error of approximation (RMSEA), which represents closeness of fit.[22] Missing data were handled using full information maximum likelihood estimation, which computes parameter estimates on the basis of all available data under the assumption that data are missing at random. However, attrition due to mortality or dropout can violate this assumption. In addition to the models described earlier, we jointly modeled loss to follow-up with the latent growth curve. Logistic regression was used to estimate the probability of loss to follow-up at wave t based on the individual's age and

For peer review only - http://bmjopen.bmj.com/site/about/guidelines.xhtml

wealth at baseline and walking speed score at *t – 1*; results are presented in Supplementary Tables 1 and 2. Estimates from this model showed that loss to follow-up did not affect estimates of mean levels (intercept and slope) of BMI and WC. Models were fitted using Mplus version 7.

In order to show graphically the level of BMI and WC at baseline, direction, and amount of change throughout the age range of our sample, we present aging-vector graphs of predicted BMI and WC. The aging graph revealed both trends by age of the sample at baseline and cohort specific within-person changes over time [23] in BMI and WC by wealth and were fitted using Stata 14.

Results

Baseline (2004-2005) characteristics of the samples are shown in Table 1. Overall mean age at baseline was 66, and the average BMI was 28 kg/m^2 and the average WC was 95.7cm. Approximately two third of people were cohabiting with a partner, and 20% of people were in each quintile of wealth. In the poorest wealth quintile the average wealth was £14 047 (range £-126 990 to £74 710) for the BMI sample and £12 961 (range £-126 990 to £71 500) in the WC sample. Almost one in seven people were physically inactive and current smokers, and two thirds of people reported a limiting long standing illness.

[Table 1 here]

BMI and WC age-trajectories

Table 2 shows the baseline level of BMI (28.22Kg/m^2) and WC (96.69cm) and change overtime (BMI slope 0.030 Kg/m^2 and WC slope 0.194cm) after accounting for age. The intercept value refers to the average value of members that were aged 66 at baseline, and the slope refers to the increase per each additional year of the study. The ageing-vector graphs shown in Figure 1 provide a visual summary of the results presented in Table 2. Each arrow (vector) represents the predicted BMI and WC at baseline and the 8-year change for every two-year age cohort. The horizontal axis indicates the respondent's age at the beginning and the end of the study period from wave 2 to wave 6 (2004-2005 to 2012-2013). The vertical axis indicates the respondent's predicted BMI (panel a) and WC (panel b). The

For peer review only - http://bmjopen.bmj.com/site/about/guidelines.xhtml
For peer review only

figures show that in early old age there was an increase in both BMI and WC, thereafter BMI and WC decreased steeply, from the age of 71 for BMI and 80 for WC.

[Table 2 here]

Wealth differences in BMI and WC age-trajectories

Table 3 shows parameter estimates adjusted for age (linear and quadratic), sex, cohabiting status, limiting longstanding illness, smoking and physical inactivity. In the top part of the table the values 27.06 Kg/m^2 and 100.29cm represent the estimated baseline mean BMI and WC for a man aged 66y in the richest wealth quintile with average conditions, which increased 8 years later to 27.21 Kg/m^2 (non-significant change) and 101.55cm, respectively. For an individual of same age in the poorest quintile of wealth, the mean BMI and WC at baseline was 29.21 Kg/m^2 and 104.93cm, which increased 8 years later to 29.36 Kg/m^2 and 106.19cm respectively. Because age was negatively associated both with intercept and slope, we provide an example for an individual aged 80y at baseline as representative of older old ages: the baseline average BMI and WC for men in the richest wealth quintile was 25.41 Kg/m^2 and 97.34 cm respectively, compared to 27.56 Kg/m^2 and 101.98cm for those in the poorest wealth quintile. Eight years later BMI was 24.42 Kg/m^2 and WC was 96.96cm for men in the richest wealth quintile compared to 26.57 Kg/m^2 and 101.60cm in the poorest wealth quintile.

There was no difference in BMI between men and women. However, overall WC at baseline was 11.13cm lower ($p<0.001$) in women, but women experienced a significantly higher increase over time compared to men, as indicated by the positive coefficient of sex on the slope (0.062 $p<0.05$).

[Table 3 here]

Figures 2 and 3 provide summaries of the model presented in Table 3, comparing people in the richest and poorest wealth quintiles. All variables including sex (but excluding wealth) have been centred on their overall mean; therefore, the estimates are an average for men and women, with average health

For peer review only - http://bmjopen.bmj.com/site/about/guidelines.xhtml

and lifestyle conditions. BMI showed a baseline intercept of 27.07 kg/m^2 and a change per 4 years of +0.019 kg/m^2, and WC at baseline was 94.17cm, with a 4-year change of +0.194 cm.

[Figures 2 and 3 here]

Discussion

Using a large nationally representative sample of older adults in England, we found that age (linear and quadratic) was a strong predictor of both initial levels and changes over time in body size defined by BMI and WC. Our results showed that, in early old age, both BMI and WC increased significantly over the 8-year follow-up, whereas at older ages, both BMI and WC decreased steeply. When the age-trajectories were explored by wealth, we found significant differences in initial levels of BMI and WC, with people in the poorest wealth quintile displaying significantly higher BMI and WC than those in the richest quintile. However, the rate of change in both BMI and WC did not differ significantly according to wealth, meaning that the age-trajectories of BMI and WC were parallel across wealth categories and that the socioeconomic gap did not close at older ages. At any given age, individuals in the lowest wealth group presented a disadvantage compared to their richer counterparts.

Longitudinal studies of change in BMI in older ages are limited and yielded to mixed results in particular regarding the age at which the trend reverses from positive to negative change over time.[10-12] Overall, our study is consistent with the literature that shows an increase in BMI over time from middle-age to early old age and a decrease thereafter. Some studies have found the trend to reverse from age 55,[24 25] whereas, more similar to our finding of a reversed trend from the age of 70 is a study in the Health and Retirement Survey in the US which showed that the trend reversed from the age of 67y.[10]

Accumulation of fat in the visceral region, has been shown to be particularly detrimental for cardiometabolic outcomes, as opposed to subcutaneous fat, and WC is a better measure to detect central obesity and overall body fat, compared to BMI which cannot adequately distinguish between

For peer review only - http://bmjopen.bmj.com/site/about/guidelines.xhtml

fat and lean mass. Despite the potential superiority of this marker for prediction of adverse aging outcomes, there is only limited evidence of the age-related changes in WC in older adults and how they may vary according to socioeconomic circumstances. We found a significant increase over time in WC in individuals aged up to 79, which is consistent with two longitudinal studies of individuals aged up to 75 years.[26 27] However, our study is the first to describe the decline in WC from the age of 80, as there are no studies that have explored changes in WC among individuals aged over 75 years.

The decrease in WC is delayed by 9 years after the start of decline in BMI. This potentially means that from age 70 onwards, the decrease in BMI reflects a loss of muscle mass not compensated by an increase in fat mass, whereas an age-related change in the fat distribution is reflected by a still increasing WC. A previous study in ELSA showed that underweight is associated with higher risk of frailty, and the more so as in the presence of abdominal obesity. [28] In the older old, from 80y onwards, we depicted a loss in both lean and fat body mass. An indirect public health implication of our observations is that efforts should be made to emphasize the importance of physical activity and a healthy diet both to limit muscle loss and accumulation of visceral fat at older age.

Socioeconomic differences in trajectories of body size in older adults have been explored using social class, income and/or education. Our study is the first to use wealth as an indicator of socioeconomic position, which is not directly comparable with the existing literature. Nevertheless, our results of a significant association of wealth on baseline levels but not on the rate of change in BMI are in accordance with using education in the US [10] and in a population of older Japanese.[17] However in the latter study, the trend was opposite when using income, with people with a lower income displaying lower BMI compared to the highest income categories. Comparisons with other studies on wealth differences in age-trajectories of WC was not possible, since there this is the first study to carry out such examination. The measure of wealth we used encompasses assets and net worth, carrying with it information on individuals' past circumstances, therefore it might be a better indicator of the permanent socioeconomic position of older adults, compared to income, social class and education.

For peer review only - http://bmjopen.bmj.com/site/about/guidelines.xhtml

This study has several limitations. First, data on BMI and WC before the age of 50 was not collected. Therefore we were not able to identify the period during the life course when inequalities started. Second, as both overall and central obesity are related to an increase risk of early mortality and chronic conditions, it is possible that we underestimated baseline inequalities in BMI and WC. Another limitation of our study is the proportion of missing data. In order to minimize the potential bias derived from missing data, we used full information maximum likelihood estimation. It is unlikely that attrition substantially influenced our results about wealth disparities in anthropometric measures. We used joint modelling to provide estimates of change in anthropometric measures that allow for correlation between these measures at previous waves and attrition. The results from the joint models (Supplementary Tables 1 and 2) were similar, which suggested that the missing data at random assumption was reasonable. Despite these limitations, our study has several strengths that make it unique. It is the first large nationally representative study in England to depict age-specific trajectories of BMI and WC over an 8-year period and to examine whether these differed by wealth. The latent growth curve modelling is an originality of our study compared to the literature, furthermore, both BMI and WC were assessed objectively by a trained nurse as opposed to most studies which used self-reported data. Lastly, we used a measure of socioeconomic status that is particularly relevant at older ages, since it reflects both past and current socioeconomic status.

To conclude, we have shown that adults aged 50 and above experienced a gain in both BMI and WC in early old age but the trend reversed at older old age when body size decreased steeply. People in the poorest wealth groups had overall higher initial levels of BMI and WC than their richer counterparts, however, the change over time in both BMI and WC did not differ according to wealth. Addressing socioeconomic inequalities in total and abdominal obesity and preventing weight gain is a crucial challenge at any stage of life, including at early old age. Equally important is the detection of and prevention of frailty at older old age.

For peer review only

Contributors: PZ designed and conducted the study. PZ and CL analysed the data, and wrote the manuscript.

Ethical approval: ELSA was approved by NHS Research Ethics Committees under the National Research and Ethics Service. All participants provided informed consent.

Competing interests: The authors declare no competing financial interests.

Funding: This work was supported by the National Institute of Aging (R01AG017644) and a consortium of UK government departments coordinated by the Economic and Social Research Council. The funding bodies had no role in the study design; in the collection, analysis, and interpretation of data; in the writing of the manuscript; and in the decision to submit the manuscript for publication.

Acknowledgment: The English Longitudinal Study of Ageing was developed by a team of researchers based at the University College London, NatCen Social Research, the Institute for Fiscal Studies and the University of Manchester. The data were collected by NatCen Social Research.

Data sharing: ELSA data is available free upon registration to the UK Data Archive http://data-archive.ac.uk/, codes are available directly from the authors

For peer review only

References

1. Wang YC, McPherson K, Marsh T, et al. Health and economic burden of the projected obesity trends in the USA and the UK. *Lancet* 2011;378(9793):815-25. doi: 10.1016/S0140-6736(11)60814-3
2. Wormser D, Kaptoge S, Di Angelantonio E, et al. Separate and combined associations of body-mass index and abdominal adiposity with cardiovascular disease: collaborative analysis of 58 prospective studies. *Lancet* 2011;377(9771):1085-95. doi: 10.1016/S0140-6736(11)60105-0
3. Must A, Spadano J, Coakley EH, et al. The disease burden associated with overweight and obesity. *JAMA* 1999;282(16):1523-9.
4. Zamboni M, Mazzali G, Zoico E, et al. Health consequences of obesity in the elderly: a review of four unresolved questions. *Int J Obes (Lond)* 2005;29(9):1011-29. doi: 10.1038/sj.ijo.0803005
5. Angleman SB, Harris TB, Melzer D. The role of waist circumference in predicting disability in periretirement age adults. *Int J Obes (Lond)* 2006;30(2):364-73. doi: 10.1038/sj.ijo.0803130
6. Flegal KM, Graubard BI, Williamson DF, et al. Cause-specific excess deaths associated with underweight, overweight, and obesity. *Jama-J Am Med Assoc* 2007;298(17):2028-37. doi: DOI 10.1001/jama.298.17.2028
7. Price GM, Uauy R, Breeze E, et al. Weight, shape, and mortality risk in older persons: elevated waist-hip ratio, not high body mass index, is associated with a greater risk of death. *Am J Clin Nutr* 2006;84(2):449-60.
8. Somes GW, Kritchevsky SB, Shorr RI, et al. Body mass index, weight change, and death in older adults: the systolic hypertension in the elderly program. *Am J Epidemiol* 2002;156(2):132-8.
9. Corrada MM, Kawas CH, Mozaffar F, et al. Association of body mass index and weight change with all-cause mortality in the elderly. *Am J Epidemiol* 2006;163(10):938-49. doi: 10.1093/aje/kwj114
10. Botoseneanu A, Liang J. Social stratification of body weight trajectory in middle-age and older americans: results from a 14-year longitudinal study. *J Aging Health* 2011;23(3):454-80. doi: 10.1177/0898264310385930
11. Dugravot A, Sabia S, Stringhini S, et al. Do socioeconomic factors shape weight and obesity trajectories over the transition from midlife to old age? Results from the French GAZEL cohort study. *Am J Clin Nutr* 2010;92(1):16-23. doi: 10.3945/ajcn.2010.29223
12. Kuchibhatla MN, Fillenbaum GG, Kraus WE, et al. Trajectory classes of body mass index in a representative elderly community sample. *J Gerontol A Biol Sci Med Sci* 2013;68(6):699-704. doi: 10.1093/gerona/gls215
13. Kahng SK, Dunkle RE, Jackson JS. The relationship between the trajectory of body mass index and health trajectory among older adults - Multilevel modeling analyses. *Res Aging* 2004;26(1):31-61. doi: 10.1177/0164027503258734
14. Britton KA, Massaro JM, Murabito JM, et al. Body Fat Distribution, Incident Cardiovascular Disease, Cancer, and All-Cause Mortality. *J Am Coll Cardiol* 2013;62(10):921-25. doi: 10.1016/j.jacc.2013.06.027
15. Wang Y, Beydoun MA. The obesity epidemic in the United States--gender, age, socioeconomic, racial/ethnic, and geographic characteristics: a systematic review and meta-regression analysis. *Epidemiol Rev* 2007;29:6-28. doi: 10.1093/epirev/mxm007
16. McLaren L. Socioeconomic status and obesity. *Epidemiol Rev* 2007;29:29-48. doi: 10.1093/epirev/mxm001
17. Murayama H, Liang J, Bennett JM, et al. Socioeconomic Status and the Trajectory of Body Mass Index Among Older Japanese: A Nationwide Cohort Study of 1987-2006. *J Gerontol B Psychol Sci Soc Sci* 2016;71(2):378-88. doi: 10.1093/geronb/gbu183
18. Pollack CE, Chideya S, Cubbin C, et al. Should health studies measure wealth? A systematic review. *Am J Prev Med* 2007;33(3):250-64.

For peer review only - http://bmjopen.bmj.com/site/about/guidelines.xhtml

19. Zaninotto P, Sacker A, Head J. Relationship between wealth and age trajectories of walking speed among older adults: evidence from the English Longitudinal Study of Ageing. *J Gerontol A Biol Sci Med Sci* 2013;68(12):1525-31. doi: 10.1093/gerona/glt058
20. Acciai F. The age pattern of social inequalities in health at older ages: are common measures of socio-economic status interchangeable? *Public Health* 2018;157:135-41. doi: 10.1016/j.puhe.2018.01.002 [published Online First: 2018/03/11]
21. Steptoe A, Breeze E, Banks J, et al. Cohort profile: the English longitudinal study of ageing. *Int J Epidemiol* 2013;42(6):1640-8. doi: 10.1093/ije/dys168
22. Hu LT, Bentler PM. Cutoff Criteria for Fit Indexes in Covariance Structure Analysis: Conventional Criteria Versus New Alternatives. *Struct Equ Modeling* 1999;6(1):1-55. doi: 10.1080/10705519909540118
23. Mirowsky J, Kim JY. Graphing age trajectories - Vector graphs, synthetic and virtual cohort projections, and cross-sectional profiles of depression. *Sociol Method Res* 2007;35(4):497-541. doi: 10.1177/0049124106296015
24. Feng X, Wilson A. Getting Bigger, Quicker? Gendered Socioeconomic Trajectories in Body Mass Index across the Adult Lifecourse: A Longitudinal Study of 21,403 Australians. *PLoS One* 2015;10(10):e0141499. doi: 10.1371/journal.pone.0141499
25. Insaf TZ, Shaw BA, Yucel RM, et al. Lifecourse socioeconomic position and 16 year body mass index trajectories: differences by race and sex. *Prev Med* 2014;67:17-23. doi: 10.1016/j.ypmed.2014.06.024
26. Liese AD, Doring A, Hense HW, et al. Five year changes in waist circumference, body mass index and obesity in Augsburg, Germany. *Eur J Nutr* 2001;40(6):282-88. doi: DOI 10.1007/s394-001-8357-0
27. Ebrahimi-Mameghani M, Scott JA, Der G, et al. Changes in weight and waist circumference over 9 years in a Scottish population. *Eur J Clin Nutr* 2008;62(10):1208-14. doi: 10.1038/sj.ejcn.1602839
28. Hubbard RE, Lang IA, Llewellyn DJ, et al. Frailty, body mass index, and abdominal obesity in older people. *J Gerontol A Biol Sci Med Sci* 2010;65(4):377-81. doi: 10.1093/gerona/glp186

For peer review only

Table 1. Baseline characteristics of the participants, ELSA (2004/2005)

SD standard deviation

	**BMI Sample N=7225**		**Waist circumference sample N=7416**	
	**Mean**	**SD**	**Mean**	**SD**
Age	66.2	9.4	66.3	9.6
BMI	27.9	4.9	26.7	7.3
Waist Circumference	95.5	13.0	95.7	13.2
Wealth quintiles				
Poorest	14047	23260	12961	21839
2nd wealth quintile	124194	25760	121520	25937
3 3rd wealth quintile	202418	22842	200200	122864
4th wealth quintile	300462	36636	2981110	36380
Richest	742495	683891	738069	674415
	**%**	**95% CI**	**%**	**95% CI**
Female	54.9	53.7; 56.0	55.0	53.9; 56.1
Cohabiting with a partner	67.2	66.2; 68.3	66.7	65.6; 67.8
Current smoker	14.5	13.7; 15.3	14.6	13.8; 15.4
Inactive/Sedentary	14.4	13.6; 15.2	15.1	14.3; 15.9
Limiting long standing illness	33.9	32.8; 35.0	34.5	33.4; 35.6
Diabetes	7.5	6.9; 8.1	7.6	7.0; 8.2
CHD	9.0	8.4; 9.7	9.0	8.3; 9.7

For peer review only

Table 2. Latent Growth Model of anthropometric measures by age, ELSA 2004/2005 to 2012/2013

	BMI			Waist circumference		
	Estimate	95% CI	p-value	Estimate	95% CI	p-value
Intercept	28.17	28.04; 28.30	<0.001	96.69	101.29,102.26	<0.001
Intercept variance	23.44	22.66; 23.44	<0.001	158.44	112.19,131.07	<0.001
Slope	0.030	0.019; 0.041	<0.001	0.194	0.13,0.22	<0.001
Slope variance	0.094	0.080; 0.109	<0.001	0.270	0.07,0.48	<0.001
**Intercept regressed on:**						
Baseline age [a] linear	-0.036	-0.047;-0.025	<0.001	0.010	-0.05,0.14	0.556
Baseline age quadratic	-0.003	-0.004;-0.002	<0.001	-0.009	-0.012; -0.006	<0.001
**Slope regressed on:**						
Baseline age [a] linear	-0.006	-0.006;-0.002	<0.001	-0.009	-0.01,0.004	<0.001
Baseline age quadratic	-0.0003	-0.001; -0.000	0.005	-0.0004	-0.001; -0.000	0.029
**Model Fit**						
CFI	0.998				0.986	
TLI	0.993				0.959	
RMSEA	0.040				0.096	
N	7225				7416	

CFI = Comparative Fit Index; RMSEA = root-mean-square error of approximation; TLI = Tucker–Lewis index.

[a] Baseline age is centred at the grand mean of 66 years

For peer review only

For peer review only

Table 3. Adjusted Latent Growth Model of anthropometric measures by age and wealth, ELSA 2004/2005 to 2012/2013

	BMI			Waist circumference		
	**Estimate[a]**	**95% CI**	**p-value**	**Estimate**	**95% CI**	**p-value**
Intercept	27.06	26.77; 27.35	<0.001	100.29	99.57; 101.00	<0.001
Intercept variance	27.77	20.89; 26.65	<0.001	122.69	117.27; 131.07	<0.001
Slope	0.019	-0.004; 0.043	0.109	0.157	0.091; 0.224	<0.001
Slope variance	0.092	0.075; 0.109	<0.001	0.328	0.206; 0.450	<0.001
**Intercept regressed on:**						
Baseline age linear	-0.076	-0.089;-0.062	<0.001	-0.085	-0.118; -0.053	<0.001
Baseline age quadratic	-0.003	-0.004;-0.002	<0.001	-0.009	-0.011; -0.006	<0.001
Female	0.023	0.200; 0.245	0.843	-11.13	-11.67; -10.59	<0.001
Poorest wealth	2.150	1.779; 2.251	<0.001	4.664	3.762; 5.565	<0.001
2nd wealth quintile	1.353	1.003; 1.702	<0.001	3.068	2.216; 3.920	<0.001
3 3rd wealth quintile	1.528	1.184; 1.873	<0.001	3.066	2.227; 3.905	<0.001
4th wealth quintile	0.668	0.325; 1.011	<0.001	1.367	0.532; 2.201	<0.001
**Slope regressed on:**						
Baseline age linear	-0.006	-0.006;-0.002	<0.001	-0.009	-0.013;-0.006	<0.001
Baseline age quadratic	-0.0003	-0.001; -0.000	0.007	-0.0004	-0.001; -0.000	0.015
Female	0.000	-0.019; 0.019	0.994	0.068	0.015; 0.120	0.011
Poorest wealth	0.001	-0.031; 0.033	0.972	-0.024	-0.113; 0.065	0.600
2nd wealth quintile	0.021	-0.008; 0.049	0.157	0.036	-0.070; 0.116	0.383
3 3rd wealth quintile	0.025	-0.002; 0.053	0.074	0.056	-0.046; 0.134	0.160
4th wealth quintile	0.008	-0.019; 0.036	0.555	-0.041	-0.141; 0.035	0.291
**Model Fit**						
CFI	0.998			0.987		
TLI	0.993			0.961		
RMSEA	0.021			0.049		
N	7225			7416		

CFI = Comparative Fit Index; RMSEA = root-mean-square error of approximation; TLI = Tucker–Lewis index.

[a] Estimates centered to the mean age of 66y and adjusted for marital status, physical inactivity, smoking status and limiting long standing illness, all centered to the grand mean.

For peer review only

Figure Legends

Figure 1. Vector graph showing 8-year aging vectors of anthropometric markers, ELSA 2004-5 to 2012-13

Figure 2. Vector graph showing adjusted 8-year aging vectors of BMI by wealth, ELSA 2004-5 to 2012-13

Figure 3. Vector graph showing adjusted 8-year aging vectors of waist circumference by wealth, ELSA 2004-5 to 2012-13

For peer review only

For peer review only - http://bmjopen.bmj.com/site/about/guidelines.xhtml

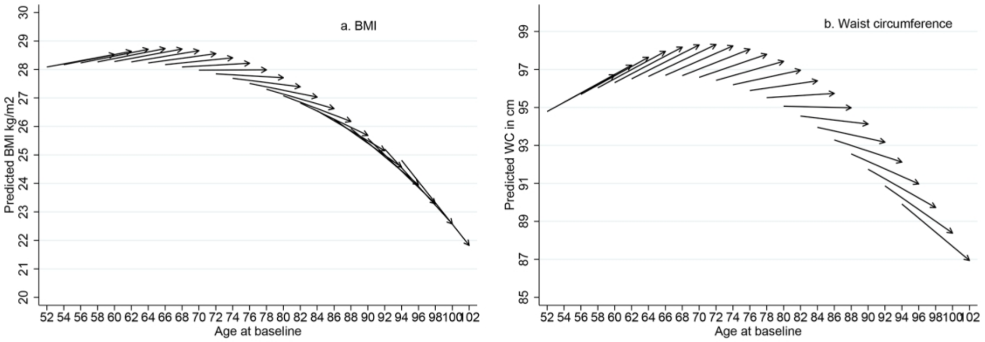

170x61mm (300 x 300 DPI)

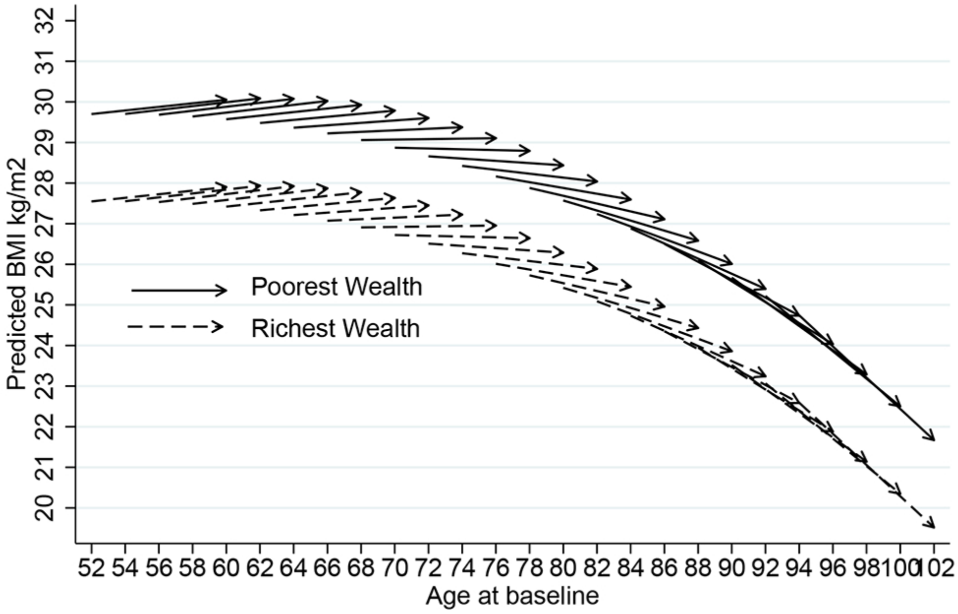

86x55mm (300 x 300 DPI)

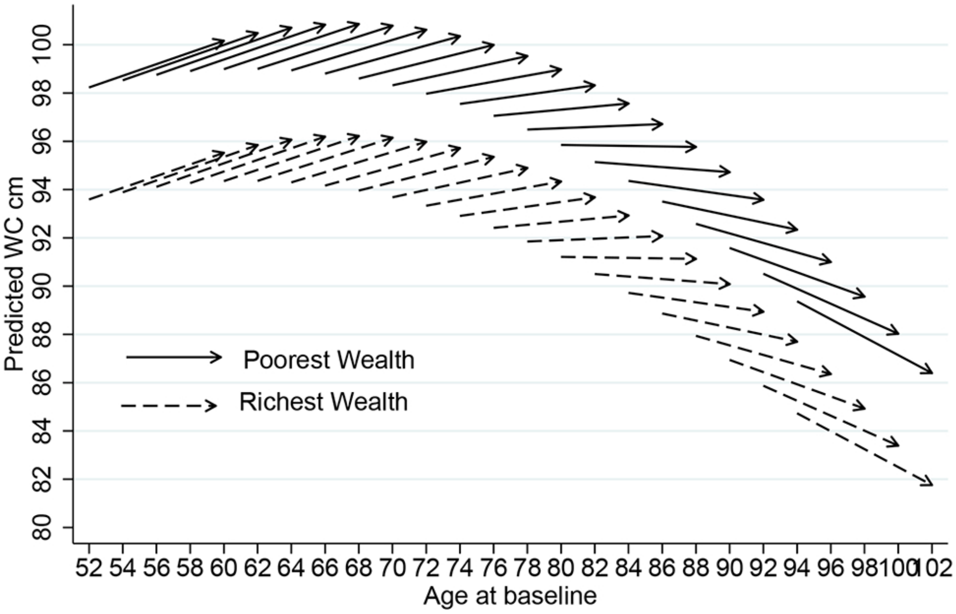

86x55mm (300 x 300 DPI)

Supplementary Tables

Socioeconomic status and trajectories of body mass index and waist circumference in older adults

Table S1 Joint Model of Attrition and Latent Growth Model of BMI with age, England 2004-5 to 2012-13

	Estimate	95% CI	p-value
**Growth parameters**			
Intercept	28.17	28.03; 28.32	<0.001
Intercept variance	22.95	22.66; 23.44	<0.001
Slope	0.031	0.018; 0.044	<0.001
Slope variance	0.079	0.047; 0.112	<0.001
**Time-invariant predictors**			
Intercept regressed on			
Baseline age	-0.036	-0.050;-0.023	<0.001
Quadratic effect of age	-0.003	-0.004;-0.002	<0.001
Slope regressed on			
Baseline age	-0.006	-0.007;-0.002	<0.001
Quadratic effect of age	-0.0003	-0.0007; -0.000	0.007
**Loss to follow up at wave 4 on**	**OR**	**95% CI**	**p-value**
BMI	1.007	0.996;1.012	0.209
Baseline age	1.042	1.036;1.049	<0.001
Quadratic effect of age	1.002	1.001;1.003	<0.001
**Loss to follow up at wave 6 on**			
BMI	1.011	1.001;1.021	0.039
Baseline age	1.063	1.057;1.021	<0.001
Quadratic effect of age	1.003	1.003;1.004	<0.001
N	7,225		

For peer review only

Table S2 Joint Model of Attrition and Latent Growth Model of waist circumference with age, England 2004-5 to 2012-13

	Estimate	95% CI	p-value
**Growth parameters**			
Intercept	96.71	96.30,97.12	<0.001
Intercept variance	157.03	150.49,163.57	<0.001
Slope	0.196	0.159,0.234	<0.001
Slope variance	0.233	0.0.92,0.374	0.001
**Time-invariant predictors**			
Intercept regressed on			
Baseline age	0.010	-0.025,0.045	0.556
Quadratic effect of age	-0.009	-0.012;-0.006	<0.001
Slope regressed on			
Baseline age	-0.009	-0.012;-0.006	<0.001
Quadratic effect of age	-0.0004	-0.0008; -0.000	0.029
**Loss to follow up at wave 4 on**	**OR**	**95% CI**	**p-value**
Waist circumference	1.007	1.003;1.011	0.001
Baseline age	1.044	1.038;1.050	<0.001
Quadratic effect of age	1.002	1.002;1.003	<0.001
**Loss to follow up at wave 6 on**			
Waist circumference	1.007	1.003;1.011	<0.001
Baseline age	1.063	1.057;1.069	<0.001
Quadratic effect of age	1.003	1.003;1.004	<0.001
N	7,416		

For peer review only

STROBE Statement—Checklist of items that should be included in reports of ***cohort studies***

	Item No	Recommendation
**Title and abstract**	1	(*a*) Indicate the study's design with a commonly used term in the title or the abstract **Pages 1 and 2**
		(*b*) Provide in the abstract an informative and balanced summary of what was done and what was found **Page 2**
**Introduction**		
Background/rationale	2	Explain the scientific background and rationale for the investigation being reported **Page 3**
Objectives	3	State specific objectives, including any prespecified hypotheses **page 3**
**Methods**		
Study design	4	Present key elements of study design early in the paper **Page 4**
Setting	5	Describe the setting, locations, and relevant dates, including periods of recruitment, exposure, follow-up, and data collection **Page 4**
Participants	6	(*a*) Give the eligibility criteria, and the sources and methods of selection of participants. Describe methods of follow-up. **Page 4**
		(*b*) For matched studies, give matching criteria and number of exposed and unexposed **NA**
Variables	7	Clearly define all outcomes, exposures, predictors, potential confounders, and effect modifiers. Give diagnostic criteria, if applicable **Pages 4-5**
Data sources/ measurement	8*	For each variable of interest, give sources of data and details of methods of assessment (measurement). Describe comparability of assessment methods if there is more than one group **Pages 4-6**
Bias	9	Describe any efforts to address potential sources of bias **NA**
Study size	10	Explain how the study size was arrived at **page 4**
Quantitative variables	11	Explain how quantitative variables were handled in the analyses. If applicable, describe which groupings were chosen and why **page 5 and 6**
Statistical methods	12	(*a*) Describe all statistical methods, including those used to control for confounding **pages 5 and 6**
		(*b*) Describe any methods used to examine subgroups and interactions **NA**
		(*c*) Explain how missing data were addressed **pages 5 and 6 and supplementary materials**
		(*d*) If applicable, explain how loss to follow-up was addressed **pages 5 and 6 and supplementary materials**
		(*e*) Describe any sensitivity analyses **pages 5 and 6 and supplementary materials**
**Results**		
Participants	13*	(a) Report numbers of individuals at each stage of study—eg numbers potentially eligible, examined for eligibility, confirmed eligible, included in the study, completing follow-up, and analysed **page 8 and 9 and Supplementary Tables 1 and 2**
		(b) Give reasons for non-participation at each stage **page 4**
		(c) Consider use of a flow diagram **NA**

For peer review only

Descriptive data	14*	(a) Give characteristics of study participants (eg demographic, clinical, social) and information on exposures and potential confounders **Table1**
		(b) Indicate number of participants with missing data for each variable of interest **Table 1**
		(c) Summarise follow-up time (eg, average and total amount) **Page 4**
Outcome data	15*	Report numbers of outcome events or summary measures over time **Page 4**
Main results	16	(*a*) Give unadjusted estimates and, if applicable, confounder-adjusted estimates and their precision (eg, 95% confidence interval). Make clear which confounders were adjusted for and why they were included **Tables 2**
		(*b*) Report category boundaries when continuous variables were categorized **NA**
		(*c*) If relevant, consider translating estimates of relative risk into absolute risk for a meaningful time period **NA**
Other analyses	17	Report other analyses done—eg analyses of subgroups and interactions, and sensitivity analyses **page 6 and Supplementary Tables 1 and 2.**
**Discussion**		
Key results	18	Summarise key results with reference to study objectives **Pages 8 and 9**
Limitations	19	Discuss limitations of the study, taking into account sources of potential bias or imprecision. Discuss both direction and magnitude of any potential bias **Page 10**
Interpretation	20	Give a cautious overall interpretation of results considering objectives, limitations, multiplicity of analyses, results from similar studies, and other relevant evidence **Page 10**
Generalisability	21	Discuss the generalisability (external validity) of the study results **Page 10**
**Other information**		
Funding	22	Give the source of funding and the role of the funders for the present study and, if applicable, for the original study on which the present article is based **Page 11**

*Give information separately for exposed and unexposed groups.

Note: An Explanation and Elaboration article discusses each checklist item and gives methodological background and published examples of transparent reporting. The STROBE checklist is best used in conjunction with this article (freely available on the Web sites of PLoS Medicine at http://www.plosmedicine.org/, Annals of Internal Medicine at http://www.annals.org/, and Epidemiology at http://www.epidem.com/). Information on the STROBE Initiative is available at http://www.strobe-statement.org.

For peer review only

BMJ Open

Socioeconomic trajectories of body mass index and waist circumference: results from the English Longitudinal Study of Ageing

Journal:	*BMJ Open*
Manuscript ID	bmjopen-2018-025309.R1
Article Type:	Research
Date Submitted by the Author:	21-Nov-2018
Complete List of Authors:	Zaninotto, Paola; UCL, Epidemiology and Public Health<br>lassale, Camille; University College London, Epidemiology and Public Health
**Primary Subject Heading**:	Epidemiology
Secondary Subject Heading:	Public health
Keywords:	body mass index, waist circumference, Trajectories, Wealth, Ageing

SCHOLARONE™
Manuscripts

Socioeconomic trajectories of body mass index and waist circumference: results from the English Longitudinal Study of Ageing

Paola Zaninotto[1], Camille Lassale[1]

[1]Department of Epidemiology and Public Health, University College London, London, UK

Corresponding author:

Paola Zaninotto, PhD

Associate Professor in Medical Statistics,

Department of Epidemiology and Public Health, University College London

1-19 Torrington Place

London, WC1E 7HB, UK

p.zaninotto@ucl.ac.uk

0044(0)2076791668

Key words: Body Mass Index, Waist circumference, Trajectories, Wealth, Aging

Licence for publication: The Corresponding Author has the right to grant on behalf of all authors and does grant on behalf of all authors, an exclusive licence (or non-exclusive for government employees) on a worldwide basis to the BMJ Publishing Group Ltd to permit this article (if accepted) to be published in JECH and any other BMJPGL products and sub-licences such use and exploit all subsidiary rights, as set out in our licence (http://group.bmj.com/products/journals/instructions-for-authors/licence-forms).

For peer review only

For peer review only - http://bmjopen.bmj.com/site/about/guidelines.xhtml

Abstract

Objectives: To explore age-trajectories of Body Mass Index (BMI) and waist circumference (WC) and to examine whether these trajectories varied by wealth.

Design: Nationally representative prospective cohort study.

Setting: Observational study of people living in England.

Participants: 7416 participants aged 52 and over of the English Longitudinal Study of Ageing (2004-2012).

Primary outcome measures: BMI and WC assessed objectively by a trained nurse.

Main exposure measure: Total non-pension household wealth quintiles defined as financial wealth, physical wealth (such as business wealth, land, or jewels), and housing wealth (primary and secondary residential housing wealth), minus debts.

Results: Using latent growth curve models we showed that BMI increased by 0.03Kg/m^2 (95%CI 0.02; 0.04 p<0.001) per year and WC by 0.18cm (0.15,0.22, p<0.001). Age (linear and quadratic) showed a negative association with BMI and WC baseline and rates of change, indicating that older individuals had smaller body sizes and that the positive rates of change flattened to eventually become negative. The decline occurred around the age of 71y for BMI and 80y for WC. Poorest wealth was significantly related to higher baseline levels of BMI (1.97Kg/m^2 95%CI:0.99; 1.55, p<0.001) and WC (4.66cm 95%CI:3.68; 2.40, p<0.001). However, no significant difference was found in the rate of change of BMI and WC by wealth, meaning that the age-trajectories of BMI and WC were parallel across wealth categories and that the socioeconomic gap did not close at older ages.

Conclusions: Older English adults showed an increase in BMI and WC over time but this trend reversed at older old age to display a sharp decrease. At any given age wealthier people had more favourable BMI and WC profile.

For peer review only

Strengths and limitations of this study

- This is the first large nationally representative study in England to depict age-specific trajectories of Body Mass Index and waist circumference over an 8-year period and to examine whether these differed by wealth.
- A trained nurse assessed Body Mass Index and waist circumference objectively.
- This study employed wealth as measure of socioeconomic status that is particularly relevant at older ages, since it reflects both past and current socioeconomic status.
- The study findings are limited to men and women aged 52 and over.

For peer review only

Introduction

There has been a marked increase in the number of obese older adults over the past decades, and the number is set to grow as the population continues to age. In the UK it is estimated that there will be an additional 11 million obese adults by 2030, of whom 3.3 million aged over 60 years.[1] The obesity epidemic has negative health consequences also in older adults where excess adiposity has been associated with chronic diseases,[2 3] disability[4 5] and mortality.[6 7]

It is increasingly recognised that a dynamic measure of body mass index (BMI) better predicts future health risks among older adults.[8 9] In older adults it has been shown that BMI increased over time up to early old age,[10 11] and declined thereafter[12 13] due to loss in muscle mass (sarcopenia).
Increasing evidence indicates differential cardiovascular risk according to adipose depots location, with increased risk observed with visceral compared to subcutaneous fat.[14]
Therefore in older adults waist circumference can be a better predictor of cardiovascular risk than BMI, and both indicators are important to monitor total and visceral adiposity.

Total and central obesity are socially patterned in developed countries with higher rates of obesity observed in lower socioeconomic groups.[15 16] However, there is limited evidence on BMI trajectories in relation to socioeconomic indicators in older adults, and results are contradictory.[10 11 17] To our knowledge, no studies from developed countries specifically reported socioeconomic trajectories of waist circumference among older adults. Furthermore, despite evidence of the importance of using wealth as an indicator of socioeconomic status of older individuals,[18-20] no studies have investigated longitudinal changes in BMI and waist circumference among older adults by wealth.

Our study has two specific aims. The first objective is to depict age-trajectories of BMI and waist circumference by analysing 8 years (2004-2005 to 2012-2013) of longitudinal data from a nationally representative sample of English adults aged 52 and over. The second objective is to examine whether these trajectories varied by wealth. To allow reliable tracking of changes over time, we use growth

For peer review only - http://bmjopen.bmj.com/site/about/guidelines.xhtml

curve models which enable studying between-person differences in within-person change by fitting fixed and random effects.[21]

Methods

The data are from three waves of the English Longitudinal Study of Ageing (ELSA),[22] where the same individuals aged 50 and older and living in private households in England, were followed and re-interviewed every 2 years. The study began in 2002-2003 (first phase of data collection referred to as wave 1) with 11 391 individuals. Data collection includes a face-to-face interview and a self-completion questionnaire at each wave and trained nurses performed visits in the participants' homes at alternative waves, to collect anthropometry measures and blood samples. For the purpose of this study, we used data from wave 2 (2004-2005), wave 4 (2008-2009) and wave 6 (2012-2013) where nurse visits took place and anthropometric measurements were taken. The sample size consisted of 7255 (5042 at wave 4 and 4256 at wave 6) for the study of BMI trajectories and 7416 (4907 at wave4 and 4147 at wave 6) for the study of waist circumference trajectories. Attrition rates are presented in Supplementary Table S1. Total follow-up time was 8 years (average 4).

Patient and public involvement statement

Patients did not participate in the design and data analysis of this study.

Anthropometric measurements

Height, weight, and waist circumference (WC) were measured during the nurse visit. Height without shoes was measured using a portable stadiometer. One measurement was taken with the informants stretching to the maximum height and the head in the Frankfort plane. Weight was measured using a portable electronic scale. Informants were asked to remove their shoes and any bulky clothing. BMI was defined as weight (kg)/height (m^2) (ranged from 14.9 to 56.2 kg/m^2). WC was defined as the midpoint between the lower rib and the upper margin of the iliac crest. Two measurements were taken using a tape with an insertion buckle at one end. Circumference was recorded to the nearest even millimetre. The mean values of the two valid measurements (the two that were the closest to each

For peer review only - http://bmjopen.bmj.com/site/about/guidelines.xhtml

other, if there were three measurements) was used in the analysis (ranged between 60.8 and 108.0 cm).

Wealth

Total non-pension household wealth, reported at the household level, was defined as financial wealth, physical wealth (such as business wealth, land, or jewels), and housing wealth (primary and secondary residential housing wealth), minus debts. The variable is excluding regular pension payments, but includes lump sums from private pension that have already been received but not yet consumed. The total score (range -126,990 to 9,319,227, mean 276,702 SD 396,453) was divided into five equal quintiles. More detailed information can be found in supplementary materials.

Covariates

We used a continuous variable for age (ranging from 52 to 101 at baseline) and a dichotomous variable for sex (0"Male" 1"Female"). From information on marital and cohabiting status we derived a dichotomous variable for cohabitation status (0"Currently living with a partner whether married or not" 1"Currently not living with a partner"). During the interview respondents were asked whether they had any longstanding illnesses, and if the illnesses limited their daily activities; responses were combined into a dichotomous variable, classifying participants as having a limiting longstanding illness or not.

Statistical analysis

To best depict reliable trajectories of anthropometric measures and to provide insight into how the rate of change relates to other variables, we used latent growth curve modelling. Growth curve modelling is specifically designed to capture change over time, by estimating latent growth factors for the baseline status (intercept) and rate of change (linear slope) over the 8-year period.[21 23-26] Latent factors representing intercept and slope components were derived from the three observations of anthropometric measures at wave 2 (baseline), wave 4, and wave 6. Factor loadings of the intercept component to all three observations were fixed to 1, and the linear slope component was defined by fixing the parameters to 0 (baseline, wave 2), 4 (wave 4), and 8 (wave 6) so that the slope parameter

For peer review only - http://bmjopen.bmj.com/site/about/guidelines.xhtml

can be interpreted as the change per year. To depict age trajectories of BMI and WC we fitted models with a linear and a quadratic term of age (non-adjusted). To answer our second research question, we tested the associations of wealth quintiles (5 categories, with 'richest wealth' as the reference category) with intercept and slope. All models included age (linear and quadratic, centred at the mean [66y]), sex, cohabiting status (binary, centred at the mean [0.51]), limiting long standing illness (binary, centred at the mean [0.67]) as covariates, all measured at baseline. For simplicity, we only present parameter estimates for age, sex and wealth. To determine the fit of the models, we employed the Comparative Fit Index (CFI), the Tucker–Lewis index (TLI), and the root-mean-square error of approximation (RMSEA), which represents closeness of fit.[27] The growth curve models (and descriptive statistics) accounted for the complex survey design and were weighted to adjust for non-response and to make the ELSA sample representative of the population of adults aged 50 and over, living in private households in England. Missing data were handled using full information maximum likelihood estimation, which computes parameter estimates on the basis of all available data under the assumption that data are missing at random. Models were fitted using Mplus version 7.

In order to show graphically the level of BMI and WC at baseline, direction, and amount of change throughout the age range of our sample, we present ageing-vector graphs[28] of predicted BMI and WC. The ageing graphs were fitted using Stata 15.

Sensitivity Analyses

We run a sensitivity analysis to test whether attrition due to mortality or dropout violated the missing at random assumption. To test for this, we jointly modelled loss to follow-up with the latent growth curve. Logistic regression was used to estimate the probability of loss to follow-up at wave t based on the individual's age and wealth at baseline and BMI or WC at $t - 1$.

We also tested for interaction terms between wealth and gender for each outcome, BMI and WC, as the impact of wealth on body size trajectory may differ by sex.

For peer review only - http://bmjopen.bmj.com/site/about/guidelines.xhtml

Results

Baseline (2004-2005) descriptive characteristics of the samples are shown in Table 1. In the "BMI" analytical sample (n=7225), overall mean age at baseline was 66, and average BMI was 27.9 kg/m^2, 53% of people were women; 70% were cohabiting with a partner, and 34% reported having a limiting long standing illness. The "Waist Circumference" analytical sample (n=7416) was essentially similar and the average WC was 95.8cm.

[Table 1 here]

BMI and WC age-trajectories

Table 2 shows the baseline level of BMI (28.19Kg/m^2) and WC (96.98cm) and change overtime (BMI slope 0.030 Kg/m^2 and WC slope 0.183cm) after accounting for age. The intercept value refers to the average value of members that were aged 66 at baseline, and the slope refers to the increase (in kg/m2 for BMI or in cm for WC) per each additional year. Age was negatively associated both with intercept and slope. The negative estimate of age on the intercept reflects a cohort effect (older generations have lower values of BMI and WC than younger generations). The negative effect of age on the slope means that the overall positive slope observed at age 66 is actually negative at older ages. The ageing-vector graphs shown in Figure 1 provide a visual summary. Each arrow (vector) represents the predicted BMI and WC at baseline and the 8-year change for every two-year age cohort. The arrows initiate at the beginning of the study period (wave 2, 2004-2005) and finish at the end of the study (wave 6, 2012-2013). The horizontal axis indicates the respondent's age. The vertical axis indicates the respondent's predicted BMI (panel a) and WC (panel b). The figures show that in early old age there was an increase in both BMI and WC, thereafter BMI and WC decreased steeply, from the age of 71 for BMI and 80 for WC. The graphs also reveal cohort differences in the trajectories of anthropometric measures, so that younger generations (born later) display greater body size indicators compared with older generations. For example, someone born in 1934, who was 70 in 2004 had a BMI of 27.9 Kg/m^2 and a WC of 96.8cm, whereas someone born in 1942 who was aged 70 in 2012 had both higher BMI and WC (BMI 28.7 Kg/m^2 and WC 98.6cm).

For peer review only - http://bmjopen.bmj.com/site/about/guidelines.xhtml

[Table 2 here]

Wealth differences in BMI and WC age-trajectories

Table 3 shows parameter estimates adjusted for age (linear and quadratic), sex, cohabiting status, limiting longstanding illness. In the top part of the table the values 27.00 Kg/m^2 and 100.09 cm represent the estimated baseline mean BMI and WC for a man aged 66y in the richest wealth quintile with average conditions, which increased 8 years later to 27.12 Kg/m^2 (non-significant change) and 101.19 cm ($p<0.001$), respectively. For an individual of same age in the poorest quintile of wealth, the mean BMI and WC at baseline was 28.97 Kg/m^2 and 104.75 cm, which increased 8 years later to 29.10 Kg/m^2 and 105.85 cm respectively. For example, for a man aged 80y at baseline: the baseline average BMI and WC if he was in the richest wealth quintile were 24.43 Kg/m^2 and 98.31cm respectively, compared to 26.40 Kg/m^2 and 102.97 cm for those in the poorest wealth quintile. Eight years later BMI was 22.34 Kg/m^2 and WC was 96.72 cm for men in the richest wealth quintile compared to 24.31 Kg/m^2 and 101.38cm in the poorest wealth quintile.

There was no difference in BMI between men and women. However, overall WC at baseline was 10.78 cm lower ($p<0.001$) in women, but women experienced a significantly higher increase over time compared to men, as indicated by the positive coefficient of sex on the slope (0.065 $p<0.05$).

[Table 3 here]

Figures 2 and 3 provide summaries of the model presented in Table 3, comparing people in the richest and poorest wealth quintiles. All variables including sex (but excluding wealth) have been centred on their overall mean; therefore, the estimates are an average for men and women, with average health and demographic conditions. BMI showed a baseline intercept of 27.09 kg/m^2 and a change per 4 years of +0.016 kg/m^2, and WC at baseline was 94.30 cm, with a 4-year change of +0.172 cm.

[Figures 2 and 3 here]

For peer review only - http://bmjopen.bmj.com/site/about/guidelines.xhtml

Sensitivity analyses

In supplementary Tables 2 and 3 we present the results of the joint model of loss to follow-up with the latent growth curve. Estimates from this model showed that loss to follow-up did not affect estimates of mean levels (intercept and slope) of BMI and WC.

Interaction terms between wealth and gender on the intercepts and slopes of BMI and WC were not significant (Supplementary Table 4). Furthermore, the results of multi-group growth curve models showed that trajectories of BMI and WC with wealth do not differ by gender (supplementary Tables 5 and 6), the model fit indicates that this model does not fit the data better than the model with gender as covariate.

Discussion

Using a large nationally representative sample of older adults in England, we found that age (linear and quadratic) was a strong predictor of both initial levels and changes over time in body size defined by BMI and WC. Our results showed that, in early old age, both BMI and WC increase significantly over the 8-year follow-up, whereas at older ages, both BMI and WC decrease steeply. When the age-trajectories were explored by wealth, we found significant differences in initial levels of BMI and WC, with people in the poorest wealth quintile displaying significantly higher BMI and WC than those in the richest quintile. However, the rate of change in both BMI and WC did not differ significantly according to wealth, meaning that the age-trajectories of BMI and WC were parallel across wealth categories and that the socioeconomic gap did not close at older ages. At any given age, individuals in the lowest wealth group presented a disadvantage compared to their richer counterparts.

Longitudinal studies of change in BMI in older ages are limited and yielded to mixed results in particular regarding the age at which the trend reverses from positive to negative change over time.[10-12] Overall, our study is consistent with the literature that shows an increase in BMI over time from middle-age to early old age and a decrease thereafter. Some studies have found the trend to reverse from age 55,[29 30] whereas, an analysis in the Health and Retirement Study in the US found the trend

For peer review only - http://bmjopen.bmj.com/site/about/guidelines.xhtml

reversed from the age of 67y which is more similar to our finding of a reversed trend from the age of 70.[10]

Accumulation of fat in the visceral region, has been shown to be particularly detrimental for cardiometabolic outcomes, as opposed to subcutaneous fat, and WC is a better measure to detect central obesity and overall body fat, compared to BMI which cannot adequately distinguish between fat and lean mass. Despite the potential superiority of this marker for prediction of adverse aging outcomes, there is only limited evidence of the age-related changes in WC in older adults and how they may vary according to socioeconomic circumstances. We found a significant increase over time in WC in individuals aged up to 79, which is consistent with two longitudinal studies of individuals aged up to 75 years.[31 32] However, our study is the first to describe the decline in WC from the age of 80, as there are no studies that have explored changes in WC among individuals aged over 75 years.

The decrease in WC is delayed by 9 years after the start of decline in BMI. This potentially means that from age 70 onwards, the decrease in BMI reflects a loss of muscle mass not compensated by an increase in fat mass, whereas an age-related change in the fat distribution is reflected by a still increasing WC. A previous study in ELSA showed that underweight is associated with higher risk of frailty, and the more so as in the presence of abdominal obesity.[33] In the older old, from 80y onwards, we depicted a loss in both lean and fat body mass. An indirect public health implication of our observations is that efforts should be made to emphasize the importance of physical activity and a healthy diet both to limit muscle loss and accumulation of visceral fat at older age.

Socioeconomic differences in trajectories of body size in older adults have been explored using social class, income and/or education. Our study is the first to use wealth as an indicator of socioeconomic position, which is not directly comparable with the existing literature. Nevertheless, our results of a significant association of wealth on baseline levels but not on the rate of change in BMI are in accordance with using education in the US[10] and in a population of older Japanese.[17] However in the latter study, the trend was opposite when using income, with people with a lower income displaying lower BMI compared to the highest income categories. Comparisons with other studies on wealth

For peer review only - http://bmjopen.bmj.com/site/about/guidelines.xhtml

differences in age-trajectories of WC was not possible, since this is the first study to carry out such examination. The measure of wealth we used encompasses assets and net worth, carrying with it information on individuals' past circumstances, therefore it might be a better indicator of the permanent socioeconomic status of older adults, compared to income, social class and education. Our results uncover yet another aspect of health social inequalities, whereby disadvantaged populations are more likely to suffer from overweight but also to become frailer at older ages, with inequalities tracking over the life course rather than fading at older ages. This emphasises the need to provide programmes of weight management and lifestyle counselling, as well as frailty assessment in the disadvantaged communities. Research should focus on developing and evaluating the efficacy of such programmes.

This study has several limitations. First, data on BMI and WC before the age of 50 was not collected. Therefore we were not able to identify the period during the life course when inequalities started. Second, as both overall and central obesity are related to an increase risk of early mortality and chronic conditions, it is possible that we underestimated baseline inequalities in BMI and WC. Another limitation of our study is the proportion of missing data. In order to minimize the potential bias derived from missing data, we used full information maximum likelihood estimation. It is unlikely that attrition substantially influenced our results about wealth disparities in anthropometric measures. We used joint modelling to provide estimates of change in anthropometric measures that allow for correlation between these measures at previous waves and attrition. The results from the joint models (Supplementary Tables 1 and 2) were similar, which suggested that the missing data at random assumption was reasonable. Despite these limitations, our study has several strengths that make it unique. It is the first large nationally representative study in England to depict age-specific trajectories of BMI and WC over an 8-year period and to examine whether these differed by wealth. The latent growth curve modelling is an originality of our study compared to the literature, furthermore, both BMI and WC were assessed objectively by a trained nurse as opposed to most studies which used self-reported data. Lastly, we used a measure of socioeconomic status that is particularly relevant at older ages, since it reflects both past and current socioeconomic status.

For peer review only - http://bmjopen.bmj.com/site/about/guidelines.xhtml

To conclude, we have shown that older English adults experienced a gain over time in both BMI and WC in early old age but the trend reversed at older old age when body size decreased steeply. People in the poorest wealth groups had overall higher initial levels of BMI and WC than their richer counterparts, however, the change over time in both BMI and WC did not differ according to wealth, meaning that that the socioeconomic gap did not close at older ages. Addressing socioeconomic inequalities in total and abdominal obesity and preventing weight gain is a crucial challenge at any stage of life, including at early old age. Equally important is the detection of and prevention of frailty at older old age.

For peer review only

Contributors: PZ designed and conducted the study. PZ and CL analysed the data, and wrote the manuscript.

Ethical approval: ELSA was approved by NHS Research Ethics Committees under the National Research and Ethics Service. All participants provided informed consent.

Competing interests: The authors declare no competing financial interests.

Funding: This work was supported by the National Institute of Aging (R01AG017644) and a consortium of UK government departments coordinated by the Economic and Social Research Council. The funding bodies had no role in the study design; in the collection, analysis, and interpretation of data; in the writing of the manuscript; and in the decision to submit the manuscript for publication.

Acknowledgment: The English Longitudinal Study of Ageing was developed by a team of researchers based at the University College London, NatCen Social Research, the Institute for Fiscal Studies and the University of Manchester. The data were collected by NatCen Social Research.

Data sharing: ELSA data is available free upon registration to the UK Data Archive http://data-archive.ac.uk/, codes are available directly from the authors

For peer review only - http://bmjopen.bmj.com/site/about/guidelines.xhtml

For peer review only

References

1. Wang YC, McPherson K, Marsh T, et al. Health and economic burden of the projected obesity trends in the USA and the UK. *Lancet* 2011;378(9793):815-25. doi: 10.1016/S0140-6736(11)60814-3
2. Wormser D, Kaptoge S, Di Angelantonio E, et al. Separate and combined associations of body-mass index and abdominal adiposity with cardiovascular disease: collaborative analysis of 58 prospective studies. *Lancet* 2011;377(9771):1085-95. doi: 10.1016/S0140-6736(11)60105-0
3. Must A, Spadano J, Coakley EH, et al. The disease burden associated with overweight and obesity. *JAMA* 1999;282(16):1523-9.
4. Zamboni M, Mazzali G, Zoico E, et al. Health consequences of obesity in the elderly: a review of four unresolved questions. *Int J Obes (Lond)* 2005;29(9):1011-29. doi: 10.1038/sj.ijo.0803005
5. Angleman SB, Harris TB, Melzer D. The role of waist circumference in predicting disability in periretirement age adults. *Int J Obes (Lond)* 2006;30(2):364-73. doi: 10.1038/sj.ijo.0803130
6. Flegal KM, Graubard BI, Williamson DF, et al. Cause-specific excess deaths associated with underweight, overweight, and obesity. *Jama-J Am Med Assoc* 2007;298(17):2028-37. doi: DOI 10.1001/jama.298.17.2028
7. Price GM, Uauy R, Breeze E, et al. Weight, shape, and mortality risk in older persons: elevated waist-hip ratio, not high body mass index, is associated with a greater risk of death. *Am J Clin Nutr* 2006;84(2):449-60.
8. Somes GW, Kritchevsky SB, Shorr RI, et al. Body mass index, weight change, and death in older adults: the systolic hypertension in the elderly program. *Am J Epidemiol* 2002;156(2):132-8.
9. Corrada MM, Kawas CH, Mozaffar F, et al. Association of body mass index and weight change with all-cause mortality in the elderly. *Am J Epidemiol* 2006;163(10):938-49. doi: 10.1093/aje/kwj114
10. Botoseneanu A, Liang J. Social stratification of body weight trajectory in middle-age and older americans: results from a 14-year longitudinal study. *J Aging Health* 2011;23(3):454-80. doi: 10.1177/0898264310385930
11. Dugravot A, Sabia S, Stringhini S, et al. Do socioeconomic factors shape weight and obesity trajectories over the transition from midlife to old age? Results from the French GAZEL cohort study. *Am J Clin Nutr* 2010;92(1):16-23. doi: 10.3945/ajcn.2010.29223
12. Kuchibhatla MN, Fillenbaum GG, Kraus WE, et al. Trajectory classes of body mass index in a representative elderly community sample. *J Gerontol A Biol Sci Med Sci* 2013;68(6):699-704. doi: 10.1093/gerona/gls215
13. Kahng SK, Dunkle RE, Jackson JS. The relationship between the trajectory of body mass index and health trajectory among older adults - Multilevel modeling analyses. *Res Aging* 2004;26(1):31-61. doi: 10.1177/0164027503258734
14. Britton KA, Massaro JM, Murabito JM, et al. Body Fat Distribution, Incident Cardiovascular Disease, Cancer, and All-Cause Mortality. *J Am Coll Cardiol* 2013;62(10):921-25. doi: 10.1016/j.jacc.2013.06.027

For peer review only - http://bmjopen.bmj.com/site/about/guidelines.xhtml

15. Wang Y, Beydoun MA. The obesity epidemic in the United States--gender, age, socioeconomic, racial/ethnic, and geographic characteristics: a systematic review and meta-regression analysis. *Epidemiol Rev* 2007;29:6-28. doi: 10.1093/epirev/mxm007
16. McLaren L. Socioeconomic status and obesity. *Epidemiol Rev* 2007;29:29-48. doi: 10.1093/epirev/mxm001
17. Murayama H, Liang J, Bennett JM, et al. Socioeconomic Status and the Trajectory of Body Mass Index Among Older Japanese: A Nationwide Cohort Study of 1987-2006. *J Gerontol B Psychol Sci Soc Sci* 2016;71(2):378-88. doi: 10.1093/geronb/gbu183
18. Pollack CE, Chideya S, Cubbin C, et al. Should health studies measure wealth? A systematic review. *Am J Prev Med* 2007;33(3):250-64.
19. Zaninotto P, Sacker A, Head J. Relationship between wealth and age trajectories of walking speed among older adults: evidence from the English Longitudinal Study of Ageing. *J Gerontol A Biol Sci Med Sci* 2013;68(12):1525-31. doi: 10.1093/gerona/glt058
20. Acciai F. The age pattern of social inequalities in health at older ages: are common measures of socio-economic status interchangeable? *Public Health* 2018;157:135-41. doi: 10.1016/j.puhe.2018.01.002 [published Online First: 2018/03/11]
21. Curran PJ, Obeidat K, Losardo D. Twelve Frequently Asked Questions About Growth Curve Modeling. *J Cogn Dev* 2010;11(2):121-36. doi: 10.1080/15248371003699969 [published Online First: 2010/01/01]
22. Steptoe A, Breeze E, Banks J, et al. Cohort profile: the English longitudinal study of ageing. *Int J Epidemiol* 2013;42(6):1640-8. doi: 10.1093/ije/dys168
23. Muthén BK, ST. Longitudinal studies of achievement growth using latent variable modeling. *Learning and Individual Differences* 1998;10:79-101.
24. Bollen KC, PJ. Latent curve models: A structural equation perspective. Hoboken. NJ: Wiley 2006.
25. Singer JW, JB. Applied longitudinal data analysis: Modeling change and event occurrence. New York: Oxford University Press 2003.
26. Browne MdT, SHC. Models for learning data. In: Collins LH, JL., ed. Best methods for the analysis of change. Washington, DC: American Psychological Association Press 1991:47-78.
27. Hu LT, Bentler PM. Cutoff Criteria for Fit Indexes in Covariance Structure Analysis: Conventional Criteria Versus New Alternatives. *Struct Equ Modeling* 1999;6(1):1-55. doi: 10.1080/10705519909540118
28. Mirowsky J, Kim JY. Graphing age trajectories - Vector graphs, synthetic and virtual cohort projections, and cross-sectional profiles of depression. *Sociol Method Res* 2007;35(4):497-541. doi: 10.1177/0049124106296015
29. Feng X, Wilson A. Getting Bigger, Quicker? Gendered Socioeconomic Trajectories in Body Mass Index across the Adult Lifecourse: A Longitudinal Study of 21,403 Australians. *PLoS One* 2015;10(10):e0141499. doi: 10.1371/journal.pone.0141499
30. Insaf TZ, Shaw BA, Yucel RM, et al. Lifecourse socioeconomic position and 16 year body mass index trajectories: differences by race and sex. *Prev Med* 2014;67:17-23. doi: 10.1016/j.ypmed.2014.06.024
31. Liese AD, Doring A, Hense HW, et al. Five year changes in waist circumference, body mass index and obesity in Augsburg, Germany. *Eur J Nutr* 2001;40(6):282-88. doi: DOI 10.1007/s394-001-8357-0
32. Ebrahimi-Mameghani M, Scott JA, Der G, et al. Changes in weight and waist circumference over 9 years in a Scottish population. *Eur J Clin Nutr* 2008;62(10):1208-14. doi: 10.1038/sj.ejcn.1602839

For peer review only - http://bmjopen.bmj.com/site/about/guidelines.xhtml

33. Hubbard RE, Lang IA, Llewellyn DJ, et al. Frailty, body mass index, and abdominal obesity in older people. *J Gerontol A Biol Sci Med Sci* 2010;65(4):377-81. doi: 10.1093/gerona/glp186

For peer review only

For peer review only - http://bmjopen.bmj.com/site/about/guidelines.xhtml

Table 1. Baseline characteristics of the analytical sample, the English Longitudinal Study of Ageing 2004-2005

	BMI Sample N=7225		Waist circumference sample N=7416	
	**Mean**	**SD**	**Mean**	**SD**
Age	66.2	9.4	66.3	9.5
BMI	27.9	4.9	26.7	7.3
Waist Circumference	94.2	17	95.8	13.1
Wealth quintiles	**%**	**N**	**%**	**N**
Poorest	22.0	1445	21.5	1484
2nd wealth quintile	20.0	1447	19.9	1483
33rd wealth quintile	20.0	1443	19.9	1483
4th wealth quintile	20.0	1445	19.7	1483
Richest	19.0	1445	18.9	1483
Female	53.4	3966	53.8	4079
Cohabiting with a partner	70.2	5039	69.4	5128
Limiting long standing illness	33.8	2445	34.4	2557

SD: Standard deviation. Estimates weighted for non-response weight

For peer review only

Table 2. Latent Growth Model of anthropometric measures by age, ELSA 2004/2005 to 2012/2013

	BMI			Waist circumference		
	Estimate [a]	95% CI	p-value	Estimate [a]	95% CI	p-value
Intercept	28.19	28.06; 28.32	<0.001	96.98	96.63; 97.73	<0.001
Intercept variance	23.44	22.36; 24.52	<0.001	158.12	152.30;163.93	<0.001
Slope	0.029	0.018; 0.040	<0.001	0.183	0.151; 0.215	<0.001
Slope variance	0.094	0.069; 0.120	<0.001	0.268	0.151; 0.385	<0.001
**Intercept regressed on:**						
Baseline age [b] linear	-0.037	-0.049;-0.026	<0.001	-0.015	-0.045;0.015	0.404
Baseline age quadratic	-0.003	-0.004;-0.002	<0.001	-0.010	-0.013; -0.008	<0.001
**Slope regressed on:**						
Baseline age [b] linear	-0.006	-0.007;-0.005	<0.001	-0.009	-0.011;-0.006	<0.001
Baseline age quadratic	-0.0003	-0.001; -0.000	0.015	0.000	-0.001; 0.000	0.148
**Model Fit**						
CFI	0.986				0.978	
TLI	0.959				0.935	
RMSEA	0.096				0.080	
N	7225				7416	

CFI = Comparative Fit Index; TLI = Tucker–Lewis index; RMSEA = root-mean-square error of approximation. [a] Weighted for non-response [b] Baseline age is centred at the grand mean of 66 years

For peer review only

Table 3. Adjusted Latent Growth Model of anthropometric measures by age and wealth, ELSA 2004/2005 to 2012/2013

	BMI			Waist circumference		
	Estimate [a]	95% CI	p-value	Estimate [a]	95% CI	p-value
Intercept	27.00	26.77; 27.22	<0.001	100.09	99.57; 101.00	<0.001
Intercept variance	22.51	21.47; 23.54	<0.001	125.23	117.27; 131.07	<0.001
Slope	0.016	-0.002; 0.034	0.140	0.137	0.091; 0.224	<0.001
Slope variance	0.094	0.069; 0.118	<0.001	0.315	0.206; 0.450	<0.001
**Intercept regressed on:**						
Baseline age linear	-0.051	-0.063;-0.039	<0.001	-0.029	-0.057; -0.001	0.086
Baseline age quadratic	-0.003	-0.004;-0.002	<0.001	-0.007	-0.010; -0.005	<0.001
Female	0.173	-0.017; 0.363	0.135	-10.78	-11.25; -10.31	<0.001
Poorest wealth	1.966	0.987; 1.545	<0.001	4.660	3.681; 5.239	<0.001
2nd wealth quintile	1.266	1.003; 1.702	<0.001	3.007	2.308; 3.705	<0.001
33rd wealth quintile	1.458	1.181; 1.735	<0.001	2.916	2.229; 3.603	<0.010
4th wealth quintile	0.594	0.323; 0.864	<0.001	1.215	0.551; 1.879	<0.001
**Slope regressed on:**						
Baseline age linear	-0.006	-0.007;-0.005	<0.001	-0.010	-0.012;-0.007	<0.001
Baseline age quadratic	-0.0003	-0.001; -0.000	0.014	-0.001	0.000; 0.000	0.090
Female	-0.001	-0.014; 0.016	0.910	0.065	0.022; 0.109	0.014
Poorest wealth	0.006	-0.022; 0.035	0.723	0.002	-0.076; 0.079	0.971
2nd wealth quintile	0.024	0.000; 0.048	0.107	0.053	-0.014; 0.121	0.195
33rd wealth quintile	0.030	-0.008; 0.052	0.027	0.070	-0.008; 0.133	0.064
4th wealth quintile	0.010	-0.014; 0.016	0.465	-0.035	-0.097; 0.026	0.347
**Model Fit**						
CFI	0.994			0.985		
TLI	0.983			0.954		
RMSEA	0.020			0.049		
N	7225			7416		

CFI = Comparative Fit Index; TLI = Tucker–Lewis index; RMSEA = root-mean-square error of approximation. [a] Estimates centered to the mean age of 66y and adjusted for marital status, and limiting long standing illness, all centered to the grand mean. Weighted for non-response.

For peer review only - http://bmjopen.bmj.com/site/about/guidelines.xhtml

Figure Legends

Figure 1. Vector graph showing 8-year aging vectors of anthropometric markers, ELSA 2004-5 to 2012-13

Figure 2. Vector graph showing adjusted 8-year aging vectors of BMI by wealth, ELSA 2004-5 to 2012-13

Figure 3. Vector graph showing adjusted 8-year aging vectors of waist circumference by wealth, ELSA 2004-5 to 2012-13

For peer review only

For peer review only - http://bmjopen.bmj.com/site/about/guidelines.xhtml

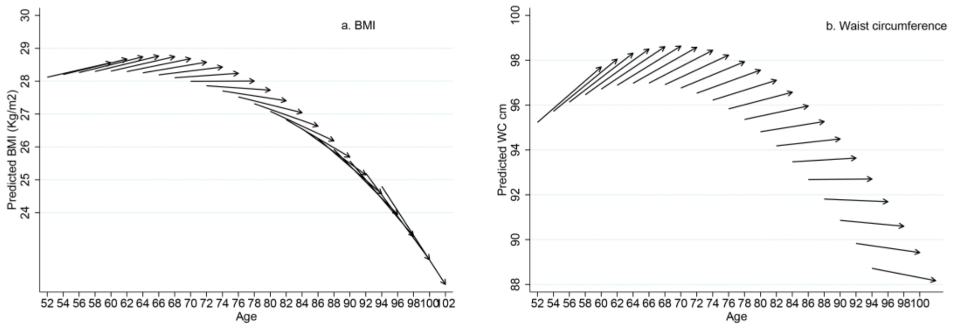

Figure 1

296x101mm (300 x 300 DPI)

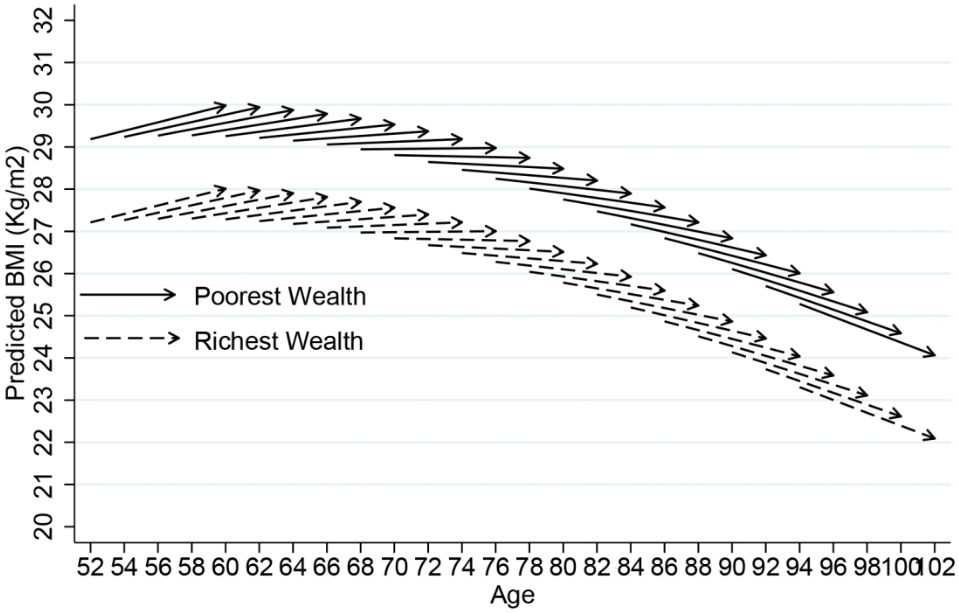

139x101mm (300 x 300 DPI)

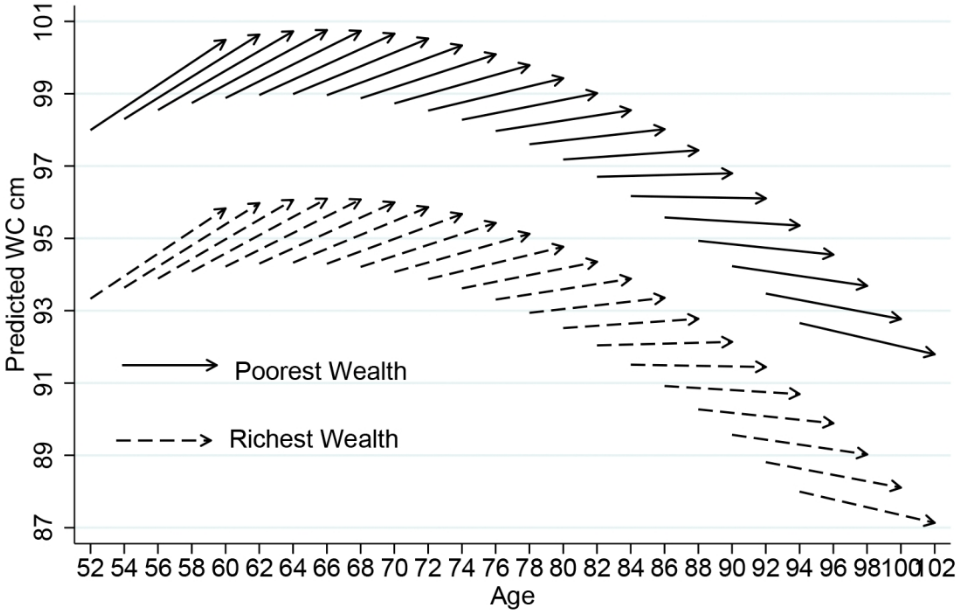

139x101mm (300 x 300 DPI)

Supplementary Materials

Socioeconomic status and trajectories of body mass index and waist circumference in older adults

Definition of Total non-pension wealth

During the face-to-face interview ELSA collects information on the financial circumstances of respondents, the questions are designed by the Institute for Fiscal Studies (IFS). From the 45 single components of income and 31 single components of wealth, an aggregate measure of wealth is derived by economists at IFS.

Total non-pension wealth: Total non-pension wealth is reported at the family level and is defined as the sum of net financial wealth, net physical wealth and net housing wealth.

Family: is a couple or a single person and any children aged below 18 they may have who are living at home.

Net financial wealth is reported at the family level and is defined as savings (interest-bearing current and deposit accounts, cash ISAs) plus investments (premium bonds, National Savings, PEPs, shares, trusts, bonds, the saving element of life insurance, shares ISAs and life insurance ISAs) and minus debt (outstanding balances on credit cards, loans, mail-order and other private debt but not including mortgages).

Net housing wealth: Net housing wealth is reported at the family level and is defined as the self-reported current value of primary housing (i.e. residential housing).

Net physical wealth: Net physical wealth is reported at the family level and is defined as wealth held in second homes, farm or business property, other business wealth, other land and other assets, such as jewellery, works of art or antiques.

For peer review only

Table S1 Sample size and attrition rates by wave of assessment

**BMI sample**	**Wave 2 (2004-2005)**	**Wave 4 (2008-2009)**	**Wave 6 (2012-2013)**
Interviewed	7,225	5,483	4,724
Dropped out		1,742	2,501
% Attrition		24.1%	34.6%
**Waist circumference sample**	**Wave 2 (2004-2005)**	**Wave 4 (2008-2009)**	**Wave 6 (2012-2013)**
Interviewed	7,416	5,609	4,821
Dropped out		1,807	2,595
Attrition		24.4%	35.0%

For peer review only

Table S2. Joint Model of Attrition and Latent Growth Model of BMI with age, England 2004-5 to 2012-13

	Estimate	95% CI	p-value
**Growth parameters**			
Intercept	28.17	28.03; 28.32	<0.001
Intercept variance	22.95	22.66; 23.44	<0.001
Slope	0.031	0.018; 0.044	<0.001
Slope variance	0.079	0.047; 0.112	<0.001
**Time-invariant predictors**			
Intercept regressed on			
Baseline age	-0.036	-0.050;-0.023	<0.001
Quadratic effect of age	-0.003	-0.004;-0.002	<0.001
Slope regressed on			
Baseline age	-0.006	-0.007;-0.002	<0.001
Quadratic effect of age	-0.0003	-0.0007; -0.000	0.007
**Loss to follow up at wave 4 on**	**OR**	**95% CI**	**p-value**
BMI	1.007	0.996;1.012	0.209
Baseline age	1.042	1.036;1.049	<0.001
Quadratic effect of age	1.002	1.001;1.003	<0.001
**Loss to follow up at wave 6 on**			
BMI	1.011	1.001;1.021	0.039
Baseline age	1.063	1.057;1.021	<0.001
Quadratic effect of age	1.003	1.003;1.004	<0.001
N	7,225		

For peer review only

Table S3 Joint Model of Attrition and Latent Growth Model of waist circumference with age, England 2004-5 to 2012-13

	Estimate	95% CI	p-value
**Growth parameters**			
Intercept	96.71	96.30,97.12	<0.001
Intercept variance	157.03	150.49,163.57	<0.001
Slope	0.196	0.159,0.234	<0.001
Slope variance	0.233	0.0.92,0.374	0.001
**Time-invariant predictors**			
Intercept regressed on			
Baseline age	0.010	-0.025,0.045	0.556
Quadratic effect of age	-0.009	-0.012;-0.006	<0.001
Slope regressed on			
Baseline age	-0.009	-0.012;-0.006	<0.001
Quadratic effect of age	-0.0004	-0.001; -0.000	0.029
**Loss to follow up at wave 4 on**	**OR**	**95% CI**	**p-value**
Waist circumference	1.007	1.003;1.011	0.001
Baseline age	1.044	1.038;1.050	<0.001
Quadratic effect of age	1.002	1.002;1.003	<0.001
**Loss to follow up at wave 6 on**			
Waist circumference	1.007	1.003;1.011	<0.001
Baseline age	1.063	1.057;1.069	<0.001
Quadratic effect of age	1.003	1.003;1.004	<0.001
N	7,416		

Table S4. P-values for interaction terms between sex and wealth in the fully adjusted Latent Growth Model of BMI and WC, ELSA 2004/2005 to 2012/2013

	BMI	WC
	**p-value**	**p-value**
**Intercept regressed on:**		
Poorest wealth & Sex Interaction	<0.010	<0.001
2nd wealth quintile & Sex Interaction	<0.010	<0.001
3^{3rd} wealth quintile & Sex Interaction	<0.010	<0.050
4th wealth quintile & Sex Interaction	<0.010	<0.050
Overall significance	<0.010	<0.010
**Slope regressed on:**		
Poorest wealth & Sex Interaction	0.081	0.039
2nd wealth quintile & Sex Interaction	0.956	0.313
3^{3rd} wealth quintile & Sex Interaction	0.256	0.775
4th wealth quintile & Sex Interaction	0.633	0.876
Overall significance	0.184	0.152

Adjusted for marital status, and limiting long standing illness. Weighted for non-response.

For peer review only

Table S5. Adjusted Sex-specific Latent Growth Model of BMI by age and wealth, ELSA 2004/2005 to 2012/2013

	Men (N=3259)			Women (N=3966)		
	**Estimate[a]**	**95% CI**	**p-value**	**Estimate[a]**	**95% CI**	**p-value**
Intercept	27.50	27.24; 27.77	<0.001	27.00	26.31; 26.91	<0.001
Intercept variance	18.38	16.98; 19.79	<0.001	25.82	24.33; 27.30	<0.001
Slope	0.003	-0.019; 0.025	0.808	0.016	0.004; 0.049	0.055
Slope variance	0.094	0.057; 0.131	<0.001	0.092	0.060; 0.125	<0.001
**Intercept regressed on:**						
Baseline age linear	-0.060	-0.076;-0.045	<0.001	-0.043	-0.061;-0.026	<0.001
Baseline age quadratic	-0.001	-0.003;-0.001	0.098	-0.004	-0.005;-0.002	<0.001
Poorest wealth	0.920	0.487; 1.352	<0.001	2.950	2.494; 3.406	<0.001
2nd wealth quintile	0.526	0.146; 0.905	<0.050	2.029	1.630; 2.429	<0.001
33rd wealth quintile	0.703	0.337; 1.068	<0.010	2.242	1.834; 2.650	<0.001
4th wealth quintile	0.030	-0.325; 0.386	0.888	1.174	0.772; 1.575	<0.001
**Slope regressed on:**						
Baseline age linear	-0.005	-0.006;-0.005	<0.001	-0.007	-0.009;-0.006	<0.001
Baseline age quadratic	0.000	0.000; 0.000	0.526	-0.0003	-0.001; -0.000	0.026
Poorest wealth	0.040	-0.001; 0.079	0.092	-0.020	-0.060; 0.019	0.402
2nd wealth quintile	0.021	-0.013; 0.054	0.308	0.022	-0.012; 0.055	0.282
33rd wealth quintile	0.046	0.014; 0.078	0.019	0.015	-0.015; 0.045	0.418
4th wealth quintile	0.012	-0.014; 0.037	0.417	0.003	-0.026; 0.031	0.884
**Model Fit**						
CFI		0.994				
TLI		0.982				
RMSEA		0.020				

CFI = Comparative Fit Index; TLI = Tucker–Lewis index; RMSEA = root-mean-square error of approximation.
[a] Estimates centered to the mean age of 66y and adjusted for marital status, and limiting long standing illness, all centered to the grand mean. Weighted for non-response.

For peer review only

Table S6. Adjusted Sex-specific Latent Growth Model of Waist circumference by age and wealth, ELSA 2004/2005 to 2012/2013

	Men (N=3337)			Women (N=4079)		
	**Estimate[a]**	**95% CI**	**p-value**	**Estimate**	**95% CI**	**p-value**
Intercept	100.87	100.15; 101.58	<0.001	88.45	88.45; 101.00	<0.001
Intercept variance	114.98	107.95; 122.01	<0.001	132.77	132.27; 131.07	<0.001
Slope	0.114	0.049; 0.180	<0.010	0.231	0.091; 0.224	<0.001
Slope variance	0.281	0.130; 0.433	<0.010	0.330	0.206; 0.450	<0.001
**Intercept regressed on:**						
Baseline age linear	-0.021	-0.060; -0.018	0.372	-0.038	-0.057; -0.001	0.120
Baseline age quadratic	-0.005	-0.008; -0.001	<0.050	-0.009	-0.010; -0.005	<0.001
Poorest wealth	2.562	1.437; 3.688	<0.001	6.167	3.681; 5.239	<0.001
2nd wealth quintile	1.674	0.689; 2.659	<0.010	4.321	2.308; 3.705	<0.001
3 3rd wealth quintile	1.946	0.973; 2.920	<0.010	3.971	2.229; 3.603	<0.001
4th wealth quintile	0.297	0.627; 1.220	0.597	2.150	0.551; 1.879	<0.001
**Slope regressed on:**						
Baseline age linear	-0.011	-0.015;-0.007	<0.001	-0.009	-0.012;-0.007	<0.001
Baseline age quadratic	-0.001	-0.001; -0.000	0.019	0.000	0.000; 0.000	0.575
Poorest wealth	0.014	-0.001; 0.228	0.103	-0.090	-0.076; 0.079	0.157
2nd wealth quintile	0.095	0.000; 0.190	0.098	0.011	-0.014; 0.121	0.846
3 3rd wealth quintile	0.077	-0.012; 0.166	0.156	0.055	-0.008; 0.133	0.298
4th wealth quintile	-0.029	-0.113; 0.056	0.577	-0.041	-0.097; 0.026	0.045
**Model Fit**						
CFI			0.981			
TLI			0.942			
RMSEA			0.052			

CFI = Comparative Fit Index; TLI = Tucker–Lewis index; RMSEA = root-mean-square error of approximation.
[a] Estimates centered to the mean age of 66y and adjusted for marital status, and limiting long standing illness, all centered to the grand mean. Weighted for non-response.

For peer review only

STROBE Statement—Checklist of items that should be included in reports of ***cohort studies***

	Item No	Recommendation
**Title and abstract**	1	(*a*) Indicate the study's design with a commonly used term in the title or the abstract **Pages 1 and 2**
		(*b*) Provide in the abstract an informative and balanced summary of what was done and what was found **Page 2**
**Introduction**		
Background/rationale	2	Explain the scientific background and rationale for the investigation being reported **Page 3**
Objectives	3	State specific objectives, including any prespecified hypotheses **page 3**
**Methods**		
Study design	4	Present key elements of study design early in the paper **Page 4**
Setting	5	Describe the setting, locations, and relevant dates, including periods of recruitment, exposure, follow-up, and data collection **Page 4**
Participants	6	(*a*) Give the eligibility criteria, and the sources and methods of selection of participants. Describe methods of follow-up. **Page 4**
		(*b*) For matched studies, give matching criteria and number of exposed and unexposed **NA**
Variables	7	Clearly define all outcomes, exposures, predictors, potential confounders, and effect modifiers. Give diagnostic criteria, if applicable **Pages 5-6. Supplementary materials**
Data sources/ measurement	8*	For each variable of interest, give sources of data and details of methods of assessment (measurement). Describe comparability of assessment methods if there is more than one group **Pages 4-6**
Bias	9	Describe any efforts to address potential sources of bias **NA**
Study size	10	Explain how the study size was arrived at **page 4**
Quantitative variables	11	Explain how quantitative variables were handled in the analyses. If applicable, describe which groupings were chosen and why **page 5 and 6**
Statistical methods	12	(*a*) Describe all statistical methods, including those used to control for confounding **pages 6 and 7**
		(*b*) Describe any methods used to examine subgroups and interactions **NA**
		(*c*) Explain how missing data were addressed **pages 6 and 7 and supplementary materials**
		(*d*) If applicable, explain how loss to follow-up was addressed **pages 6 and 7 and supplementary materials**
		(*e*) Describe any sensitivity analyses **page 7 and supplementary materials**
**Results**		
Participants	13*	(a) Report numbers of individuals at each stage of study—eg numbers potentially eligible, examined for eligibility, confirmed eligible, included in the study, completing follow-up, and analysed **page 5 and Supplementary Tables 1- 3**
		(b) Give reasons for non-participation at each stage **page 5**
		(c) Consider use of a flow diagram **NA**

For peer review only - http://bmjopen.bmj.com/site/about/guidelines.xhtml

Descriptive data	14*	(a) Give characteristics of study participants (eg demographic, clinical, social) and information on exposures and potential confounders **Table1**
		(b) Indicate number of participants with missing data for each variable of interest **Table 1**
		(c) Summarise follow-up time (eg, average and total amount) **Page 5**
Outcome data	15*	Report numbers of outcome events or summary measures over time **Page 4**
Main results	16	(*a*) Give unadjusted estimates and, if applicable, confounder-adjusted estimates and their precision (eg, 95% confidence interval). Make clear which confounders were adjusted for and why they were included **Tables 2**
		(*b*) Report category boundaries when continuous variables were categorized **NA**
		(*c*) If relevant, consider translating estimates of relative risk into absolute risk for a meaningful time period **NA**
Other analyses	17	Report other analyses done—eg analyses of subgroups and interactions, and sensitivity analyses **page 8 and Supplementary Tables 1-6.**
**Discussion**		
Key results	18	Summarise key results with reference to study objectives **Pages 8 and 9**
Limitations	19	Discuss limitations of the study, taking into account sources of potential bias or imprecision. Discuss both direction and magnitude of any potential bias **Page 11**
Interpretation	20	Give a cautious overall interpretation of results considering objectives, limitations, multiplicity of analyses, results from similar studies, and other relevant evidence **Page 11**
Generalisability	21	Discuss the generalisability (external validity) of the study results **Page 12**
**Other information**		
Funding	22	Give the source of funding and the role of the funders for the present study and, if applicable, for the original study on which the present article is based **Page 13**

*Give information separately for exposed and unexposed groups.

Note: An Explanation and Elaboration article discusses each checklist item and gives methodological background and published examples of transparent reporting. The STROBE checklist is best used in conjunction with this article (freely available on the Web sites of PLoS Medicine at http://www.plosmedicine.org/, Annals of Internal Medicine at http://www.annals.org/, and Epidemiology at http://www.epidem.com/). Information on the STROBE Initiative is available at http://www.strobe-statement.org.

For peer review only

For peer review only - http://bmjopen.bmj.com/site/about/guidelines.xhtml

BMJ Open

Socioeconomic trajectories of body mass index and waist circumference: results from the English Longitudinal Study of Ageing

Journal:	*BMJ Open*
Manuscript ID	bmjopen-2018-025309.R2
Article Type:	Research
Date Submitted by the Author:	18-Jan-2019
Complete List of Authors:	Zaninotto, Paola; UCL, Epidemiology and Public Health<br>lassale, Camille; University College London, Epidemiology and Public Health
**Primary Subject Heading**:	Epidemiology
Secondary Subject Heading:	Public health
Keywords:	body mass index, waist circumference, Trajectories, Wealth, Ageing

SCHOLARONE™
Manuscripts

Socioeconomic trajectories of body mass index and waist circumference: results from the English Longitudinal Study of Ageing

Paola Zaninotto[1], Camille Lassale[1]

[1]Department of Epidemiology and Public Health, University College London, London, UK

Corresponding author:

Paola Zaninotto, PhD

Associate Professor in Medical Statistics,

Department of Epidemiology and Public Health, University College London

1-19 Torrington Place

London, WC1E 7HB, UK

p.zaninotto@ucl.ac.uk

0044(0)2076791668

Key words: Body Mass Index, Waist circumference, Trajectories, Wealth, Aging

Licence for publication: The Corresponding Author has the right to grant on behalf of all authors and does grant on behalf of all authors, an exclusive licence (or non-exclusive for government employees) on a worldwide basis to the BMJ Publishing Group Ltd to permit this article (if accepted) to be published in JECH and any other BMJPGL products and sub-licences such use and exploit all subsidiary rights, as set out in our licence (http://group.bmj.com/products/journals/instructions-for-authors/licence-forms).

For peer review only - http://bmjopen.bmj.com/site/about/guidelines.xhtml

Abstract

Objectives: To explore age-trajectories of Body Mass Index (BMI) and waist circumference (WC) and to examine whether these trajectories varied by wealth.

Design: Nationally representative prospective cohort study.

Setting: Observational study of people living in England.

Participants: 7416 participants aged 52 and over of the English Longitudinal Study of Ageing (2004-2012).

Primary outcome measures: BMI and WC assessed objectively by a trained nurse.

Main exposure measure: Total non-pension household wealth quintiles defined as financial wealth, physical wealth (such as business wealth, land, or jewels), and housing wealth (primary and secondary residential housing wealth), minus debts.

Results: Using latent growth curve models we showed that BMI increased by 0.03Kg/m^2 (95%CI 0.02; 0.04 p<0.001) per year and WC by 0.18cm **(**0.15,0.22, p<0.001). Age (linear and quadratic) showed a negative association with BMI and WC baseline and rates of change, indicating that older individuals had smaller body sizes and that the positive rates of change flattened to eventually become negative. The decline occurred around the age of 71y for BMI and 80y for WC. Poorest wealth was significantly related to higher baseline levels of BMI (1.97Kg/m^2 95%CI:0.99; 1.55, p<0.001) and WC (4.66cm 95%CI:3.68; 2.40, p<0.001). However, no significant difference was found in the rate of change of BMI and WC by wealth, meaning that the age-trajectories of BMI and WC were parallel across wealth categories and that the socioeconomic gap did not close at older ages.

Conclusions: Older English adults showed an increase in BMI and WC over time but this trend reversed at older old age to display a sharp decrease. At any given age wealthier people had more favourable BMI and WC profile.

For peer review only

Strengths and limitations of this study

- This is the first large nationally representative study in England to depict age-specific trajectories of Body Mass Index and waist circumference over an 8-year period and to examine whether these differed by wealth.
- A trained nurse assessed Body Mass Index and waist circumference objectively.
- This study employed wealth as measure of socioeconomic status that is particularly relevant at older ages, since it reflects both past and current socioeconomic status.
- The study findings are limited to men and women aged 52 and over.

For peer review only

For peer review only - http://bmjopen.bmj.com/site/about/guidelines.xhtml

Introduction

There has been a marked increase in the number of obese older adults over the past decades, and the number is set to grow as the population continues to age. In the UK it is estimated that there will be an additional 11 million obese adults by 2030, of whom 3.3 million aged over 60 years.[1] The obesity epidemic has negative health consequences also in older adults where excess adiposity has been associated with chronic diseases,[2 3] disability[4 5] and mortality.[6 7]

It is increasingly recognised that a dynamic measure of body mass index (BMI) better predicts future health risks among older adults.[8 9] In older adults it has been shown that BMI increased over time up to early old age,[10 11] and declined thereafter[12 13] due to loss in muscle mass (sarcopenia). Increasing evidence indicates differential cardiovascular risk according to adipose depots location, with increased risk observed with visceral compared to subcutaneous fat.[14]
Therefore in older adults waist circumference can be a better predictor of cardiovascular risk than BMI, and both indicators are important to monitor total and visceral adiposity.

Total and central obesity are socially patterned in developed countries with higher rates of obesity observed in lower socioeconomic groups.[15 16] However, there is limited evidence on BMI trajectories in relation to socioeconomic indicators in older adults, and results are contradictory.[10 11 17] To our knowledge, no studies from developed countries specifically reported socioeconomic trajectories of waist circumference among older adults. Furthermore, despite evidence of the importance of using wealth as an indicator of socioeconomic status of older individuals,[18-20] no studies have investigated longitudinal changes in BMI and waist circumference among older adults by wealth.

Our study has two specific aims. The first objective is to depict age-trajectories of BMI and waist circumference by analysing 8 years (2004-2005 to 2012-2013) of longitudinal data from a nationally representative sample of English adults aged 52 and over. The second objective is to examine whether these trajectories varied by wealth. To allow reliable tracking of changes over time, we use growth

For peer review only - http://bmjopen.bmj.com/site/about/guidelines.xhtml

curve models which enable studying between-person differences in within-person change by fitting fixed and random effects.[21]

Methods

The data are from three waves of the English Longitudinal Study of Ageing (ELSA),[22] where the same individuals aged 50 and older and living in private households in England, were followed and re-interviewed every 2 years. The study began in 2002-2003 (first phase of data collection referred to as wave 1) with 11 391 individuals. Data collection includes a face-to-face interview and a self-completion questionnaire at each wave and trained nurses performed visits in the participants' homes at alternative waves, to collect anthropometry measures and blood samples. For the purpose of this study, we used data from wave 2 (2004-2005), wave 4 (2008-2009) and wave 6 (2012-2013) where nurse visits took place and anthropometric measurements were taken. The sample size consisted of 7255 (5042 at wave 4 and 4256 at wave 6) for the study of BMI trajectories and 7416 (4907 at wave4 and 4147 at wave 6) for the study of waist circumference trajectories. Attrition rates are presented in Supplementary Table S1. Total follow-up time was 8 years (average 4).

Patient and public involvement statement

Patients did not participate in the design and data analysis of this study.

Anthropometric measurements

Height, weight, and waist circumference (WC) were measured during the nurse visit. Height without shoes was measured using a portable stadiometer. One measurement was taken with the informants stretching to the maximum height and the head in the Frankfort plane. Weight was measured using a portable electronic scale. Informants were asked to remove their shoes and any bulky clothing. BMI was defined as weight (kg)/height (m^2) (ranged from 14.9 to 56.2 kg/m^2). WC was defined as the midpoint between the lower rib and the upper margin of the iliac crest. Two measurements were taken using a tape with an insertion buckle at one end. Circumference was recorded to the nearest even millimetre. The mean values of the two valid measurements (the two that were the closest to each

For peer review only - http://bmjopen.bmj.com/site/about/guidelines.xhtml

other, if there were three measurements) was used in the analysis (ranged between 60.8 and 108.0 cm).

Wealth

Total non-pension household wealth, reported at the household level, was defined as financial wealth, physical wealth (such as business wealth, land, or jewels), and housing wealth (primary and secondary residential housing wealth), minus debts. The variable is excluding regular pension payments, but includes lump sums from private pension that have already been received but not yet consumed. The total score (range -126,990 to 9,319,227, mean 276,702 SD 396,453) was divided into five equal quintiles. More detailed information can be found in supplementary materials.

Covariates

We used a continuous variable for age (ranging from 52 to 101 at baseline) and a dichotomous variable for sex (0"Male" 1"Female"). From information on marital and cohabiting status we derived a dichotomous variable for cohabitation status (0"Currently living with a partner whether married or not" 1"Currently not living with a partner"). During the interview respondents were asked whether they had any longstanding illnesses, and if the illnesses limited their daily activities; responses were combined into a dichotomous variable, classifying participants as having a limiting longstanding illness or not.

Statistical analysis

To best depict reliable trajectories of anthropometric measures and to provide insight into how the rate of change relates to other variables, we used latent growth curve modelling. Growth curve modelling is specifically designed to capture change over time, by estimating latent growth factors for the baseline status (intercept) and rate of change (linear slope) over the 8-year period.[21 23-26] Latent factors representing intercept and slope components were derived from the three observations of anthropometric measures at wave 2 (baseline), wave 4, and wave 6. Factor loadings of the intercept component to all three observations were fixed to 1, and the linear slope component was defined by fixing the parameters to 0 (baseline, wave 2), 4 (wave 4), and 8 (wave 6) so that the slope parameter

For peer review only - http://bmjopen.bmj.com/site/about/guidelines.xhtml

can be interpreted as the change per year. To depict age trajectories of BMI and WC we fitted models with a linear and a quadratic term of age (non-adjusted). To answer our second research question, we tested the associations of wealth quintiles (treated as categorical in the models with 'richest wealth' as the reference category) with intercept and slope. All models included age (linear and quadratic, centred at the mean [66y]), sex (treated as binary), cohabiting status (treated as binary, centred at the mean [0.51]), limiting long standing illness (treated as binary, centred at the mean [0.67]) as covariates, all measured at baseline. To ease interpretation of estimates for age (linear and quadratic) we used a 10 years increment in age, instead of single years. For simplicity, we only present parameter estimates for age, sex and wealth. To determine the fit of the models, we employed the Comparative Fit Index (CFI), the Tucker–Lewis index (TLI), and the root-mean-square error of approximation (RMSEA), which represents closeness of fit.[27] The growth curve models (and descriptive statistics) accounted for the complex survey design and were weighted to adjust for non-response and to make the ELSA sample representative of the population of adults aged 50 and over, living in private households in England. Missing data were handled using full information maximum likelihood estimation, which computes parameter estimates on the basis of all available data under the assumption that data are missing at random. Models were fitted using Mplus version 7.

In order to show graphically the level of BMI and WC at baseline, direction, and amount of change throughout the age range of our sample, we present ageing-vector graphs[28] of predicted BMI and WC. The ageing graphs were fitted using Stata 15.

Sensitivity Analyses

We run a sensitivity analysis to test whether attrition due to mortality or dropout violated the missing at random assumption. To test for this, we jointly modelled loss to follow-up with the latent growth curve. Logistic regression was used to estimate the probability of loss to follow-up at wave t based on the individual's age and wealth at baseline and BMI or WC at $t - 1$.

We also tested for interaction terms between wealth and gender for each outcome, BMI and WC, as the impact of wealth on body size trajectory may differ by sex.

For peer review only - http://bmjopen.bmj.com/site/about/guidelines.xhtml

Results

Baseline (2004-2005) descriptive characteristics of the samples are shown in Table 1. In the "BMI" analytical sample (n=7225), overall mean age at baseline was 66, and average BMI was 27.9 kg/m^2, 53% of people were women; 70% were cohabiting with a partner, and 34% reported having a limiting long standing illness. The "Waist Circumference" analytical sample (n=7416) was essentially similar and the average WC was 95.8cm.

[Table 1 here]

BMI and WC age-trajectories

Table 2 shows the baseline level of BMI (28.19Kg/m^2) and WC (96.98cm) and change overtime (BMI slope 0.030 Kg/m^2 and WC slope 0.183cm) after accounting for age. The intercept value refers to the average value of members that were aged 66 at baseline, and the slope refers to the increase (in kg/m2 for BMI or in cm for WC) per each additional year of the study. Age was negatively associated both with intercept and slope. The negative estimate of age on the intercept reflects a cohort effect (older generations have lower values of BMI and WC than younger generations). The negative effect of age on the slope means that the overall positive slope observed at age 66 is actually negative at older ages. The ageing-vector graphs shown in Figure 1 provide a visual summary. Each arrow (vector) represents the predicted BMI and WC at baseline and the 8-year change for every two-year age cohort. The arrows initiate at the beginning of the study period (wave 2, 2004-2005) and finish at the end of the study (wave 6, 2012-2013). The horizontal axis indicates the respondent's age. The vertical axis indicates the respondent's predicted BMI (panel a) and WC (panel b). The figures show that in early old age there was an increase in both BMI and WC, thereafter BMI and WC decreased steeply, from the age of 71 for BMI and 80 for WC. The graphs also reveal cohort differences in the trajectories of anthropometric measures, so that younger generations (born later) display greater body size indicators compared with older generations. For example, someone born in 1934, who was 70 in

For peer review only - http://bmjopen.bmj.com/site/about/guidelines.xhtml

2004 had a BMI of 27.9 Kg/m^2 and a WC of 96.8cm, whereas someone born in 1942 who was aged 70 in 2012 had both higher BMI and WC (BMI 28.7 Kg/m^2 and WC 98.6cm).

[Table 2 here]

Wealth differences in BMI and WC age-trajectories

Table 3 shows parameter estimates adjusted for age (linear and quadratic), sex, cohabiting status, limiting longstanding illness. In the top part of the table the values 27.00 Kg/m^2 and 100.09 cm represent the estimated baseline mean BMI and WC for a man aged 66y in the richest wealth quintile with average conditions, which increased 8 years later to 27.12 Kg/m^2 (non-significant change) and 101.19 cm ($p<0.001$), respectively. For an individual of same age in the poorest quintile of wealth, the mean BMI and WC at baseline was 28.97 Kg/m^2 and 104.75 cm, which increased 8 years later to 29.10 Kg/m^2 and 105.85 cm respectively. For example, for a man aged 80y at baseline: the baseline average BMI and WC if he was in the richest wealth quintile were 24.43 Kg/m^2 and 98.31cm respectively, compared to 26.40 Kg/m^2 and 102.97 cm for those in the poorest wealth quintile. Eight years later BMI was 22.34 Kg/m^2 and WC was 96.72 cm for men in the richest wealth quintile compared to 24.31 Kg/m^2 and 101.38cm in the poorest wealth quintile.

There was no difference in BMI between men and women. However, overall WC at baseline was 10.78 cm lower ($p<0.001$) in women, but women experienced a significantly higher increase over time compared to men, as indicated by the positive coefficient of sex on the slope (0.065 $p<0.05$).

[Table 3 here]

Figures 2 and 3 provide summaries of the model presented in Table 3, comparing people in the richest and poorest wealth quintiles. All variables including sex (but excluding wealth) have been centred on their overall mean; therefore, the estimates are an average for men and women, with average health and demographic conditions. BMI showed a baseline intercept of 27.09 kg/m^2 and a change per 4 years of +0.016 kg/m^2, and WC at baseline was 94.30 cm, with a 4-year change of +0.172 cm.

For peer review only - http://bmjopen.bmj.com/site/about/guidelines.xhtml

[Figures 2 and 3 here]

Sensitivity analyses

In supplementary Tables 2 and 3 we present the results of the joint model of loss to follow-up with the latent growth curve. Estimates from this model showed that loss to follow-up did not affect estimates of mean levels (intercept and slope) of BMI and WC.

Interaction terms between wealth and gender on the intercepts and slopes of BMI and WC were not significant (Supplementary Table 4). Furthermore, the results of multi-group growth curve models showed that trajectories of BMI and WC with wealth do not differ by gender (supplementary Tables 5 and 6), the model fit indicates that this model does not fit the data better than the model with gender as covariate.

For peer review only

Discussion

Using a large nationally representative sample of older adults in England, we found that age (linear and quadratic) was a strong predictor of both initial levels and changes over time in body size defined by BMI and WC. Our results showed that, in early old age, both BMI and WC increase significantly over the 8-year follow-up, whereas at older ages, both BMI and WC decrease steeply. When the age-trajectories were explored by wealth, we found significant differences in initial levels of BMI and WC, with people in the poorest wealth quintile displaying significantly higher BMI and WC than those in the richest quintile. However, the rate of change in both BMI and WC did not differ significantly according to wealth, meaning that the age-trajectories of BMI and WC were parallel across wealth categories and that the socioeconomic gap did not close at older ages. At any given age, individuals in the lowest wealth group presented a disadvantage compared to their richer counterparts.

Longitudinal studies of change in BMI in older ages are limited and yielded to mixed results in particular regarding the age at which the trend reverses from positive to negative change over time.[10-12] Overall, our study is consistent with the literature that shows an increase in BMI over time from

middle-age to early old age and a decrease thereafter. Some studies have found the trend to reverse from age 55,[29 30] whereas, an analysis in the Health and Retirement Study in the US found the trend reversed from the age of 67y which is more similar to our finding of a reversed trend from the age of 70.[10]

Accumulation of fat in the visceral region, has been shown to be particularly detrimental for cardiometabolic outcomes, as opposed to subcutaneous fat, and WC is a better measure to detect central obesity and overall body fat, compared to BMI which cannot adequately distinguish between fat and lean mass. Despite the potential superiority of this marker for prediction of adverse aging outcomes, there is only limited evidence of the age-related changes in WC in older adults and how they may vary according to socioeconomic circumstances. We found a significant increase over time in WC in individuals aged up to 79, which is consistent with two longitudinal studies of individuals aged up to 75 years.[31 32] However, our study is the first to describe the decline in WC from the age of 80, as there are no studies that have explored changes in WC among individuals aged over 75 years.

The decrease in WC is delayed by 9 years after the start of decline in BMI. This potentially means that from age 70 onwards, the decrease in BMI reflects a loss of muscle mass not compensated by an increase in fat mass, whereas an age-related change in the fat distribution is reflected by a still increasing WC. A previous study in ELSA showed that underweight is associated with higher risk of frailty, and the more so as in the presence of abdominal obesity.[33] In the older old, from 80y onwards, we depicted a loss in both lean and fat body mass. An indirect public health implication of our observations is that efforts should be made to emphasize the importance of physical activity and a healthy diet both to limit muscle loss and accumulation of visceral fat at older age.

Socioeconomic differences in trajectories of body size in older adults have been explored using social class, income and/or education. Our study is the first to use wealth as an indicator of socioeconomic position, which is not directly comparable with the existing literature. Nevertheless, our results of a significant association of wealth on baseline levels but not on the rate of change in BMI are in accordance with using education in the US[10] and in a population of older Japanese.[17] However in the

For peer review only - http://bmjopen.bmj.com/site/about/guidelines.xhtml

latter study, the trend was opposite when using income, with people with a lower income displaying lower BMI compared to the highest income categories. Comparisons with other studies on wealth differences in age-trajectories of WC was not possible, since this is the first study to carry out such examination. The measure of wealth we used encompasses assets and net worth, carrying with it information on individuals' past circumstances, therefore it might be a better indicator of the permanent socioeconomic status of older adults, compared to income, social class and education. Our results uncover yet another aspect of health social inequalities, whereby disadvantaged populations are more likely to suffer from overweight but also to become frailer at older ages, with inequalities tracking over the life course rather than fading at older ages. This emphasises the need to provide programmes of weight management and lifestyle counselling, as well as frailty assessment in the disadvantaged communities. Research should focus on developing and evaluating the efficacy of such programmes.

This study has several limitations. First, data on BMI and WC before the age of 50 was not collected. Therefore we were not able to identify the period during the life course when inequalities started. Second, as both overall and central obesity are related to an increase risk of early mortality and chronic conditions, it is possible that we underestimated baseline inequalities in BMI and WC. Another limitation of our study is the proportion of missing data. In order to minimize the potential bias derived from missing data, we used full information maximum likelihood estimation. It is unlikely that attrition substantially influenced our results about wealth disparities in anthropometric measures. We used joint modelling to provide estimates of change in anthropometric measures that allow for correlation between these measures at previous waves and attrition. The results from the joint models (Supplementary Tables 1 and 2) were similar, which suggested that the missing data at random assumption was reasonable. Despite these limitations, our study has several strengths that make it unique. It is the first large nationally representative study in England to depict age-specific trajectories of BMI and WC over an 8-year period and to examine whether these differed by wealth. The latent growth curve modelling is an originality of our study compared to the literature, furthermore, both BMI and WC were assessed objectively by a trained nurse as opposed to most

For peer review only - http://bmjopen.bmj.com/site/about/guidelines.xhtml
For peer review only

studies which used self-reported data. Lastly, we used a measure of socioeconomic status that is particularly relevant at older ages, since it reflects both past and current socioeconomic status.

To conclude, we have shown that older English adults experienced a gain over time in both BMI and WC in early old age but the trend reversed at older old age when body size decreased steeply. People in the poorest wealth groups had overall higher initial levels of BMI and WC than their richer counterparts, however, the change over time in both BMI and WC did not differ according to wealth, meaning that that the socioeconomic gap did not close at older ages. Addressing socioeconomic inequalities in total and abdominal obesity and preventing weight gain is a crucial challenge at any stage of life, including at early old age. Equally important is the detection of and prevention of frailty at older old age.

Contributors: PZ designed and conducted the study. PZ and CL analysed the data, and wrote the manuscript.

Ethical approval: ELSA was approved by NHS Research Ethics Committees under the National Research and Ethics Service. All participants provided informed consent.

Competing interests: The authors declare no competing financial interests.

Funding: This work was supported by the National Institute of Aging (R01AG017644) and a consortium of UK government departments coordinated by the Economic and Social Research Council. The funding bodies had no role in the study design; in the collection, analysis, and interpretation of data; in the writing of the manuscript; and in the decision to submit the manuscript for publication.

Acknowledgment: The English Longitudinal Study of Ageing was developed by a team of researchers based at the University College London, NatCen Social Research, the Institute for Fiscal Studies and the University of Manchester. The data were collected by NatCen Social Research.

For peer review only

Data sharing: ELSA data is available free upon registration to the UK Data Archive http://data-archive.ac.uk/, codes are available directly from the authors

For peer review only

References

1. Wang YC, McPherson K, Marsh T, et al. Health and economic burden of the projected obesity trends in the USA and the UK. *Lancet* 2011;378(9793):815-25. doi: 10.1016/S0140-6736(11)60814-3
2. Wormser D, Kaptoge S, Di Angelantonio E, et al. Separate and combined associations of body-mass index and abdominal adiposity with cardiovascular disease: collaborative analysis of 58 prospective studies. *Lancet* 2011;377(9771):1085-95. doi: 10.1016/S0140-6736(11)60105-0
3. Must A, Spadano J, Coakley EH, et al. The disease burden associated with overweight and obesity. *JAMA* 1999;282(16):1523-9.
4. Zamboni M, Mazzali G, Zoico E, et al. Health consequences of obesity in the elderly: a review of four unresolved questions. *Int J Obes (Lond)* 2005;29(9):1011-29. doi: 10.1038/sj.ijo.0803005
5. Angleman SB, Harris TB, Melzer D. The role of waist circumference in predicting disability in periretirement age adults. *Int J Obes (Lond)* 2006;30(2):364-73. doi: 10.1038/sj.ijo.0803130
6. Flegal KM, Graubard BI, Williamson DF, et al. Cause-specific excess deaths associated with underweight, overweight, and obesity. *Jama-J Am Med Assoc* 2007;298(17):2028-37. doi: DOI 10.1001/jama.298.17.2028
7. Price GM, Uauy R, Breeze E, et al. Weight, shape, and mortality risk in older persons: elevated waist-hip ratio, not high body mass index, is associated with a greater risk of death. *Am J Clin Nutr* 2006;84(2):449-60.
8. Somes GW, Kritchevsky SB, Shorr RI, et al. Body mass index, weight change, and death in older adults: the systolic hypertension in the elderly program. *Am J Epidemiol* 2002;156(2):132-8.
9. Corrada MM, Kawas CH, Mozaffar F, et al. Association of body mass index and weight change with all-cause mortality in the elderly. *Am J Epidemiol* 2006;163(10):938-49. doi: 10.1093/aje/kwj114
10. Botoseneanu A, Liang J. Social stratification of body weight trajectory in middle-age and older americans: results from a 14-year longitudinal study. *J Aging Health* 2011;23(3):454-80. doi: 10.1177/0898264310385930
11. Dugravot A, Sabia S, Stringhini S, et al. Do socioeconomic factors shape weight and obesity trajectories over the transition from midlife to old age? Results from the French GAZEL cohort study. *Am J Clin Nutr* 2010;92(1):16-23. doi: 10.3945/ajcn.2010.29223
12. Kuchibhatla MN, Fillenbaum GG, Kraus WE, et al. Trajectory classes of body mass index in a representative elderly community sample. *J Gerontol A Biol Sci Med Sci* 2013;68(6):699-704. doi: 10.1093/gerona/gls215
13. Kahng SK, Dunkle RE, Jackson JS. The relationship between the trajectory of body mass index and health trajectory among older adults - Multilevel modeling analyses. *Res Aging* 2004;26(1):31-61. doi: 10.1177/0164027503258734
14. Britton KA, Massaro JM, Murabito JM, et al. Body Fat Distribution, Incident Cardiovascular Disease, Cancer, and All-Cause Mortality. *J Am Coll Cardiol* 2013;62(10):921-25. doi: 10.1016/j.jacc.2013.06.027

For peer review only - http://bmjopen.bmj.com/site/about/guidelines.xhtml

15. Wang Y, Beydoun MA. The obesity epidemic in the United States--gender, age, socioeconomic, racial/ethnic, and geographic characteristics: a systematic review and meta-regression analysis. *Epidemiol Rev* 2007;29:6-28. doi: 10.1093/epirev/mxm007
16. McLaren L. Socioeconomic status and obesity. *Epidemiol Rev* 2007;29:29-48. doi: 10.1093/epirev/mxm001
17. Murayama H, Liang J, Bennett JM, et al. Socioeconomic Status and the Trajectory of Body Mass Index Among Older Japanese: A Nationwide Cohort Study of 1987-2006. *J Gerontol B Psychol Sci Soc Sci* 2016;71(2):378-88. doi: 10.1093/geronb/gbu183
18. Pollack CE, Chideya S, Cubbin C, et al. Should health studies measure wealth? A systematic review. *Am J Prev Med* 2007;33(3):250-64.
19. Zaninotto P, Sacker A, Head J. Relationship between wealth and age trajectories of walking speed among older adults: evidence from the English Longitudinal Study of Ageing. *J Gerontol A Biol Sci Med Sci* 2013;68(12):1525-31. doi: 10.1093/gerona/glt058
20. Acciai F. The age pattern of social inequalities in health at older ages: are common measures of socio-economic status interchangeable? *Public Health* 2018;157:135-41. doi: 10.1016/j.puhe.2018.01.002 [published Online First: 2018/03/11]
21. Curran PJ, Obeidat K, Losardo D. Twelve Frequently Asked Questions About Growth Curve Modeling. *J Cogn Dev* 2010;11(2):121-36. doi: 10.1080/15248371003699969 [published Online First: 2010/01/01]
22. Steptoe A, Breeze E, Banks J, et al. Cohort profile: the English longitudinal study of ageing. *Int J Epidemiol* 2013;42(6):1640-8. doi: 10.1093/ije/dys168
23. Muthén BK, ST. Longitudinal studies of achievement growth using latent variable modeling. *Learning and Individual Differences* 1998;10:79-101.
24. Bollen KC, PJ. Latent curve models: A structural equation perspective. Hoboken. NJ: Wiley 2006.
25. Singer JW, JB. Applied longitudinal data analysis: Modeling change and event occurrence. New York: Oxford University Press 2003.
26. Browne MdT, SHC. Models for learning data. In: Collins LH, JL., ed. Best methods for the analysis of change. Washington, DC: American Psychological Association Press 1991:47-78.
27. Hu LT, Bentler PM. Cutoff Criteria for Fit Indexes in Covariance Structure Analysis: Conventional Criteria Versus New Alternatives. *Struct Equ Modeling* 1999;6(1):1-55. doi: 10.1080/10705519909540118
28. Mirowsky J, Kim JY. Graphing age trajectories - Vector graphs, synthetic and virtual cohort projections, and cross-sectional profiles of depression. *Sociol Method Res* 2007;35(4):497-541. doi: 10.1177/0049124106296015
29. Feng X, Wilson A. Getting Bigger, Quicker? Gendered Socioeconomic Trajectories in Body Mass Index across the Adult Lifecourse: A Longitudinal Study of 21,403 Australians. *PLoS One* 2015;10(10):e0141499. doi: 10.1371/journal.pone.0141499
30. Insaf TZ, Shaw BA, Yucel RM, et al. Lifecourse socioeconomic position and 16 year body mass index trajectories: differences by race and sex. *Prev Med* 2014;67:17-23. doi: 10.1016/j.ypmed.2014.06.024
31. Liese AD, Doring A, Hense HW, et al. Five year changes in waist circumference, body mass index and obesity in Augsburg, Germany. *Eur J Nutr* 2001;40(6):282-88. doi: DOI 10.1007/s394-001-8357-0
32. Ebrahimi-Mameghani M, Scott JA, Der G, et al. Changes in weight and waist circumference over 9 years in a Scottish population. *Eur J Clin Nutr* 2008;62(10):1208-14. doi: 10.1038/sj.ejcn.1602839

For peer review only

33. Hubbard RE, Lang IA, Llewellyn DJ, et al. Frailty, body mass index, and abdominal obesity in older people. *J Gerontol A Biol Sci Med Sci* 2010;65(4):377-81. doi: 10.1093/gerona/glp186

For peer review only

Table 1. Baseline characteristics of the participants included in the samples used to model trajectories of body mass index (BMI) and waist circumference (WC) over 8 years, the English Longitudinal Study of Ageing 2004-2005

	BMI Sample N=7225		Waist circumference sample N=7416	
	**Mean**	**SD**	**Mean**	**SD**
Age	66.2	9.4	66.3	9.5
BMI	27.9	4.9	26.7	7.3
Waist Circumference	94.2	17	95.8	13.1
Wealth quintiles	**%**	**N**	**%**	**N**
Poorest	22.0	1445	21.5	1484
2nd wealth quintile	20.0	1447	19.9	1483
33rd wealth quintile	20.0	1443	19.9	1483
4th wealth quintile	20.0	1445	19.7	1483
Richest	19.0	1445	18.9	1483
Female	53.4	3966	53.8	4079
Cohabiting with a partner	70.2	5039	69.4	5128
Limiting long standing illness	33.8	2445	34.4	2557

SD: Standard deviation. Estimates weighted for non-response weight

For peer review only

For peer review only - http://bmjopen.bmj.com/site/about/guidelines.xhtml

Table 2. Latent Growth Model of anthropometric measures by age, ELSA 2004/2005 to 2012/2013

	BMI			Waist circumference		
	Estimate [a]	95% CI	p-value	Estimate [a]	95% CI	p-value
Intercept	28.19	28.06; 28.32	<0.001	96.98	96.63; 97.73	<0.001
Intercept variance	23.44	22.36; 24.52	<0.001	158.12	152.30;163.93	<0.001
Slope	0.029	0.018; 0.040	<0.001	0.183	0.151; 0.215	<0.001
Slope variance	0.094	0.069; 0.120	<0.001	0.268	0.151; 0.385	<0.001
**Intercept regressed on:**						
Baseline age[b] linear	-0.372	-0.486;-0.258	<0.001	-0.153	-0.453;0.148	0.404
Baseline age[b] quadratic	-0.276	-0.378;-0.174	<0.001	-1.042	-1.300; -0.780	<0.001
**Slope regressed on:**						
Baseline age[b] linear	-0.061	-0.072;-0.051	<0.001	-0.086	-0.114;-0.059	<0.001
Baseline age[b] quadratic	-0.017	-0.028; -0.006	0.015	-0.028	-0.061; 0.004	0.148
**Model Fit**						
CFI	0.986				0.978	
TLI	0.959				0.935	
RMSEA	0.096				0.080	
N	7225				7416	

CFI = Comparative Fit Index; TLI = Tucker–Lewis index; RMSEA = root-mean-square error of approximation. [a] Weighted for non-response [b] Baseline age is centred at the grand mean of 66 years and presented in 10years increment

For peer review only

Table 3. Adjusted Latent Growth Model of anthropometric measures by age and wealth, ELSA 2004/2005 to 2012/2013

	BMI			Waist circumference		
	Estimate [a]	95% CI	p-value	Estimate [a]	95% CI	p-value
Intercept	27.00	26.77; 27.22	<0.001	100.09	99.57; 101.00	<0.001
Intercept variance	22.51	21.47; 23.54	<0.001	125.23	117.27; 131.07	<0.001
Slope	0.016	-0.002; 0.034	0.140	0.137	0.091; 0.224	<0.001
Slope variance	0.094	0.069; 0.118	<0.001	0.315	0.206; 0.450	<0.001
**Intercept regressed on:**						
Baseline age[b] linear	-0.511	-0.628;-0.394	<0.001	-0.293	-0.574; -0.013	0.086
Baseline age[b] quadratic	-0.256	-0.357;-0.155	<0.001	-0.750	-0.981; -0.518	<0.001
Female	0.173	-0.017; 0.363	0.135	-10.78	-11.25; -10.31	<0.001
Poorest wealth	1.966	0.987; 1.545	<0.001	4.660	3.681; 5.239	<0.001
2nd wealth quintile	1.266	1.003; 1.702	<0.001	3.007	2.308; 3.705	<0.001
33rd wealth quintile	1.458	1.181; 1.735	<0.001	2.916	2.229; 3.603	<0.010
4th wealth quintile	0.594	0.323; 0.864	<0.001	1.215	0.551; 1.879	<0.001
**Slope regressed on:**						
Baseline age[b] linear	-0.062	-0.072;-0.052	<0.001	-0.097	-0.125;-0.068	<0.001
Baseline age[b] quadratic	-0.017	-0.029; -0.006	0.014	-0.033	-0.065; 0.001	0.090
Female	-0.001	-0.014; 0.016	0.910	0.065	0.022; 0.109	0.014
Poorest wealth	0.006	-0.022; 0.035	0.723	0.002	-0.076; 0.079	0.971
2nd wealth quintile	0.024	0.000; 0.048	0.107	0.053	-0.014; 0.121	0.195
33rd wealth quintile	0.030	-0.008; 0.052	0.027	0.070	-0.008; 0.133	0.064
4th wealth quintile	0.010	-0.014; 0.016	0.465	-0.035	-0.097; 0.026	0.347
**Model Fit**						
CFI	0.994			0.985		
TLI	0.983			0.954		
RMSEA	0.020			0.049		
N	7225			7416		

CFI = Comparative Fit Index; TLI = Tucker–Lewis index; RMSEA = root-mean-square error of approximation. [a] Estimates adjusted for marital status, and limiting long standing illness, all centered to the grand mean.

[b] Baseline age is centred at the grand mean of 66 years and presented in 10years increment. Weighted for non-response.

For peer review only - http://bmjopen.bmj.com/site/about/guidelines.xhtml

Figure Legends

Figure 1. Vector graph showing 8-year aging vectors of anthropometric markers, ELSA 2004-5 to 2012-13

Figure 2. Vector graph showing adjusted 8-year aging vectors of BMI by wealth, ELSA 2004-5 to 2012-13

Figure 3. Vector graph showing adjusted 8-year aging vectors of waist circumference by wealth, ELSA 2004-5 to 2012-13

For peer review only

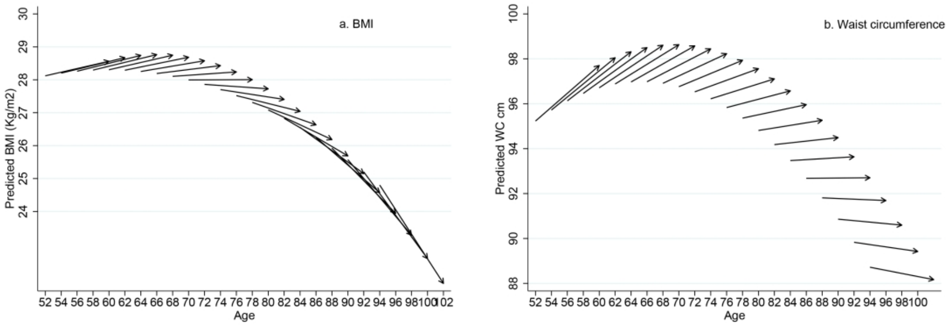

Figure 1

296x101mm (300 x 300 DPI)

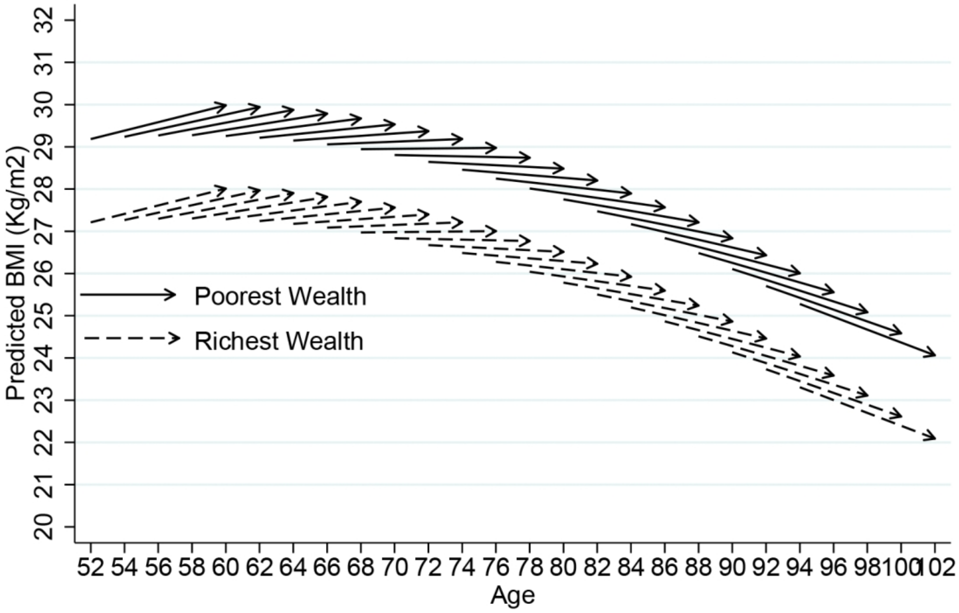

139x101mm (300 x 300 DPI)

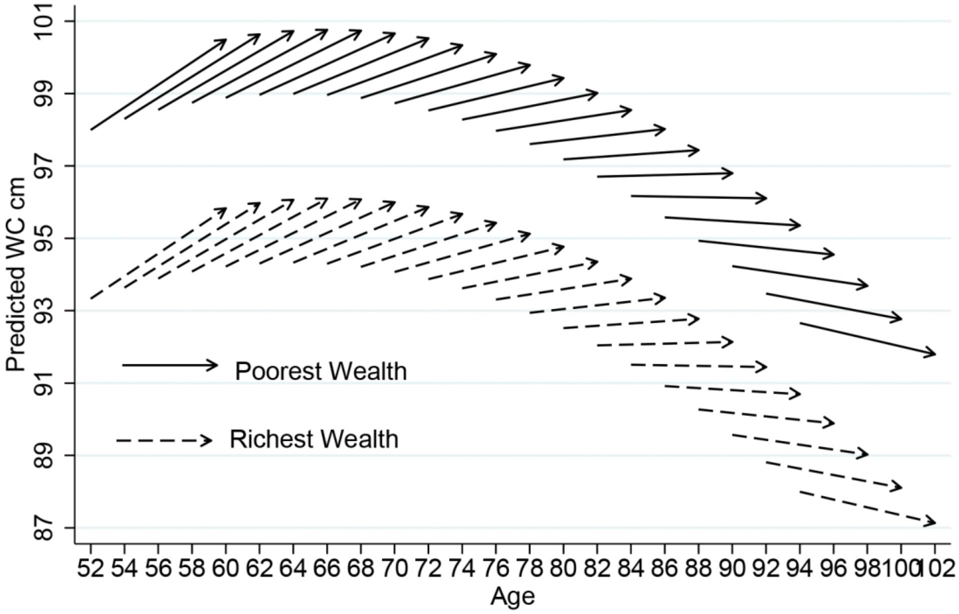

139x101mm (300 x 300 DPI)

Supplementary Materials

Socioeconomic status and trajectories of body mass index and waist circumference in older adults

Definition of Total non-pension wealth

During the face-to-face interview ELSA collects information on the financial circumstances of respondents, the questions are designed by the Institute for Fiscal Studies (IFS). From the 45 single components of income and 31 single components of wealth, an aggregate measure of wealth is derived by economists at IFS.

Total non-pension wealth: Total non-pension wealth is reported at the family level and is defined as the sum of net financial wealth, net physical wealth and net housing wealth.

Family: is a couple or a single person and any children aged below 18 they may have who are living at home.

Net financial wealth is reported at the family level and is defined as savings (interest-bearing current and deposit accounts, cash ISAs) plus investments (premium bonds, National Savings, PEPs, shares, trusts, bonds, the saving element of life insurance, shares ISAs and life insurance ISAs) and minus debt (outstanding balances on credit cards, loans, mail-order and other private debt but not including mortgages).

Net housing wealth: Net housing wealth is reported at the family level and is defined as the self-reported current value of primary housing (i.e. residential housing).

Net physical wealth: Net physical wealth is reported at the family level and is defined as wealth held in second homes, farm or business property, other business wealth, other land and other assets, such as jewellery, works of art or antiques.

For peer review only

Table S1 Sample size and attrition rates by wave of assessment

**BMI sample**	**Wave 2 (2004-2005)**	**Wave 4 (2008-2009)**	**Wave 6 (2012-2013)**
Interviewed	7,225	5,483	4,724
Dropped out		1,742	2,501
% Attrition		24.1%	34.6%
**Waist circumference sample**	**Wave 2 (2004-2005)**	**Wave 4 (2008-2009)**	**Wave 6 (2012-2013)**
Interviewed	7,416	5,609	4,821
Dropped out		1,807	2,595
Attrition		24.4%	35.0%

For peer review only

Table S2. Joint Model of Attrition and Latent Growth Model of BMI with age, England 2004-5 to 2012-13

	Estimate	95% CI	p-value
**Growth parameters**			
Intercept	28.17	28.03; 28.32	<0.001
Intercept variance	22.95	22.66; 23.44	<0.001
Slope	0.031	0.018; 0.044	<0.001
Slope variance	0.079	0.047; 0.112	<0.001
**Time-invariant predictors**			
Intercept regressed on			
Baseline age[a]	-0.362	-0.474;-0.250	<0.001
Quadratic effect of age[a]	-0.274	-0.370;-0.178	<0.001
Slope regressed on			
Baseline age[a]	-0.060	-0.070;-0.050	<0.001
Quadratic effect of age[a]	-0.018	-0.029; -0.007	0.007
**Loss to follow up at wave 4 on**	**OR**	**95% CI**	**p-value**
BMI	1.007	0.996;1.012	0.209
Baseline age[a]	1.515	1.439;1.596	<0.001
Quadratic effect of age[a]	1.228	1.172;1.288	<0.001
**Loss to follow up at wave 6 on**			
BMI	1.011	1.001;1.021	0.039
Baseline age[a]	1.842	1.754;1.934	<0.001
Quadratic effect of age[a]	1.368	1.299;1.440	<0.001
N	7,225		

[a] Baseline age is centred at the grand mean of 66 years and presented in 10years increment

For peer review only

Table S3 Joint Model of Attrition and Latent Growth Model of waist circumference with age, England 2004-5 to 2012-13

	Estimate	95% CI	p-value
**Growth parameters**			
Intercept	96.71	96.30,97.12	<0.001
Intercept variance	157.03	150.49,163.57	<0.001
Slope	0.196	0.159,0.234	<0.001
Slope variance	0.233	0.0.92,0.374	0.001
**Time-invariant predictors**			
Intercept regressed on			
Baseline age[a]	0.128	-0.248, 0.468	0.556
Quadratic effect of age[a]	-0.009	-0.012;-0.006	<0.001
Slope regressed on			
Baseline age[a]	-0.089	-0.120;-0.058	<0.001
Quadratic effect of age[a]	-0.037	-0.030; -0.006	0.029
**Loss to follow up at wave 4 on**	**OR**	**95% CI**	**p-value**
Waist circumference	1.007	1.003;1.011	0.001
Baseline age[a]	1.538	1.349;1.705	<0.001
Quadratic effect of age[a]	1.221	1.220;1.502	<0.001
**Loss to follow up at wave 6 on**			
Waist circumference	1.007	1.003;1.011	<0.001
Baseline age[a]	1.842	1.750;1.930	<0.001
Quadratic effect of age[a]	1.349	1.339;1.420	<0.001
N	7,416		

[a] Baseline age is centred at the grand mean of 66 years and presented in 10years increment

For peer review only

Table S4. P-values for interaction terms between sex and wealth in the fully adjusted Latent Growth Model of BMI and WC, ELSA 2004/2005 to 2012/2013

	**BMI**	**WC**
	**p-value**	**p-value**
**Intercept regressed on:**		
Poorest wealth & Sex Interaction	<0.010	<0.001
2nd wealth quintile & Sex Interaction	<0.010	<0.001
3^{3rd} wealth quintile & Sex Interaction	<0.010	<0.050
4th wealth quintile & Sex Interaction	<0.010	<0.050
Overall significance	<0.010	<0.010
**Slope regressed on:**		
Poorest wealth & Sex Interaction	0.081	0.039
2nd wealth quintile & Sex Interaction	0.956	0.313
3^{3rd} wealth quintile & Sex Interaction	0.256	0.775
4th wealth quintile & Sex Interaction	0.633	0.876
Overall significance	0.184	0.152

Adjusted for marital status, and limiting long standing illness. Weighted for non-response.

For peer review only

Table S5. Adjusted Sex-specific Latent Growth Model of BMI by age and wealth, ELSA 2004/2005 to 2012/2013

	**Men (N=3259)**			**Women (N=3966)**		
	**Estimate[a]**	**95% CI**	**p-value**	**Estimate[a]**	**95% CI**	**p-value**
Intercept	27.50	27.24; 27.77	<0.001	27.00	26.31; 26.91	<0.001
Intercept variance	18.38	16.98; 19.79	<0.001	25.82	24.33; 27.30	<0.001
Slope	0.003	-0.019; 0.025	0.808	0.016	0.004; 0.049	0.055
Slope variance	0.094	0.057; 0.131	<0.001	0.092	0.060; 0.125	<0.001
**Intercept regressed on:**						
Baseline age[b] linear	-0.602	-0.756;-0.448	<0.001	-0.434	-0.608;-0.261	<0.001
Baseline age[b] quadratic	-0.131	-0.262;-0.001	0.098	-0.366	-0.509;-0.223	<0.001
Poorest wealth	0.920	0.487; 1.352	<0.001	2.950	2.494; 3.406	<0.001
2nd wealth quintile	0.526	0.146; 0.905	<0.050	2.029	1.630; 2.429	<0.001
33rd wealth quintile	0.703	0.337; 1.068	<0.010	2.242	1.834; 2.650	<0.001
4th wealth quintile	0.030	-0.325; 0.386	0.888	1.174	0.772; 1.575	<0.001
**Slope regressed on:**						
Baseline age[b] linear	-0.046	-0.060;-0.032	<0.001	-0.073	-0.088;-0.059	<0.001
Baseline age[b] quadratic	-0.006	-0.023; 0.010	0.526	-0.021	-0.021; -0.006	0.026
Poorest wealth	0.040	-0.001; 0.079	0.092	-0.020	-0.060; 0.019	0.402
2nd wealth quintile	0.021	-0.013; 0.054	0.308	0.022	-0.012; 0.055	0.282
33rd wealth quintile	0.046	0.014; 0.078	0.019	0.015	-0.015; 0.045	0.418
4th wealth quintile	0.012	-0.014; 0.037	0.417	0.003	-0.026; 0.031	0.884
**Model Fit**						
CFI		0.994				
TLI		0.982				
RMSEA		0.020				

CFI = Comparative Fit Index; TLI = Tucker–Lewis index; RMSEA = root-mean-square error of approximation.
[a] Estimates adjusted for marital status, and limiting long standing illness, all centered to the grand mean.
[b] Baseline age is centred at the grand mean of 66 years and presented in 10years increment
Weighted for non-response.

For peer review only

Table S6. Adjusted Sex-specific Latent Growth Model of Waist circumference by age and wealth, ELSA 2004/2005 to 2012/2013

	Men (N=3337)			Women (N=4079)		
	**Estimate[a]**	**95% CI**	**p-value**	**Estimate**	**95% CI**	**p-value**
Intercept	100.87	100.15; 101.58	<0.001	88.45	88.45; 101.00	<0.001
Intercept variance	114.98	107.95; 122.01	<0.001	132.77	132.27; 131.07	<0.001
Slope	0.114	0.049; 0.180	<0.010	0.231	0.091; 0.224	<0.001
Slope variance	0.281	0.130; 0.433	<0.010	0.330	0.206; 0.450	<0.001
**Intercept regressed on:**						
Baseline age[b] linear	-0.212	-0.603; -0.179	0.372	-0.382	-0.057; -0.001	0.120
Baseline age[b] quadratic	-0.474	-0.831; -0.116	<0.050	-0.922	-0.010; -0.005	<0.001
Poorest wealth	2.562	1.437; 3.688	<0.001	6.167	3.681; 5.239	<0.001
2nd wealth quintile	1.674	0.689; 2.659	<0.010	4.321	2.308; 3.705	<0.001
33rd wealth quintile	1.946	0.973; 2.920	<0.010	3.971	2.229; 3.603	<0.001
4th wealth quintile	0.297	0.627; 1.220	0.597	2.150	0.551; 1.879	<0.001
**Slope regressed on:**						
Baseline age[b] linear	-0.106	-0.145;-0.067	<0.001	-0.091	-0.131;-0.050	<0.001
Baseline age[b] quadratic	-0.061	-0.104; -0.018	0.019	-0.015	-0.060; 0.030	0.575
Poorest wealth	0.014	-0.001; 0.228	0.103	-0.090	-0.076; 0.079	0.157
2nd wealth quintile	0.095	0.000; 0.190	0.098	0.011	-0.014; 0.121	0.846
33rd wealth quintile	0.077	-0.012; 0.166	0.156	0.055	-0.008; 0.133	0.298
4th wealth quintile	-0.029	-0.113; 0.056	0.577	-0.041	-0.097; 0.026	0.045
**Model Fit**						
CFI		0.981				
TLI		0.942				
RMSEA		0.052				

CFI = Comparative Fit Index; TLI = Tucker–Lewis index; RMSEA = root-mean-square error of approximation.
[a] Estimates adjusted for marital status, and limiting long standing illness, all centered to the grand mean.
[b] Baseline age is centred at the grand mean of 66 years and presented in 10years increment.
Weighted for non-response.

For peer review only

STROBE Statement—Checklist of items that should be included in reports of ***cohort studies***

	Item No	Recommendation
**Title and abstract**	1	(*a*) Indicate the study's design with a commonly used term in the title or the abstract **Pages 1 and 2**
		(*b*) Provide in the abstract an informative and balanced summary of what was done and what was found **Page 2**
**Introduction**		
Background/rationale	2	Explain the scientific background and rationale for the investigation being reported **Page 3**
Objectives	3	State specific objectives, including any prespecified hypotheses **page 3**
**Methods**		
Study design	4	Present key elements of study design early in the paper **Page 4**
Setting	5	Describe the setting, locations, and relevant dates, including periods of recruitment, exposure, follow-up, and data collection **Page 4**
Participants	6	(*a*) Give the eligibility criteria, and the sources and methods of selection of participants. Describe methods of follow-up. **Page 4**
		(*b*) For matched studies, give matching criteria and number of exposed and unexposed **NA**
Variables	7	Clearly define all outcomes, exposures, predictors, potential confounders, and effect modifiers. Give diagnostic criteria, if applicable **Pages 5-6. Supplementary materials**
Data sources/ measurement	8*	For each variable of interest, give sources of data and details of methods of assessment (measurement). Describe comparability of assessment methods if there is more than one group **Pages 4-6**
Bias	9	Describe any efforts to address potential sources of bias **NA**
Study size	10	Explain how the study size was arrived at **page 4**
Quantitative variables	11	Explain how quantitative variables were handled in the analyses. If applicable, describe which groupings were chosen and why **page 5 and 6**
Statistical methods	12	(*a*) Describe all statistical methods, including those used to control for confounding **pages 6 and 7**
		(*b*) Describe any methods used to examine subgroups and interactions **NA**
		(*c*) Explain how missing data were addressed **pages 6 and 7 and supplementary materials**
		(*d*) If applicable, explain how loss to follow-up was addressed **pages 6 and 7 and supplementary materials**
		(*e*) Describe any sensitivity analyses **page 7 and supplementary materials**
**Results**		
Participants	13*	(a) Report numbers of individuals at each stage of study—eg numbers potentially eligible, examined for eligibility, confirmed eligible, included in the study, completing follow-up, and analysed **page 5 and Supplementary Tables 1- 3**
		(b) Give reasons for non-participation at each stage **page 5**
		(c) Consider use of a flow diagram **NA**

For peer review only

Descriptive data	14*	(a) Give characteristics of study participants (eg demographic, clinical, social) and information on exposures and potential confounders **Table1**
		(b) Indicate number of participants with missing data for each variable of interest **Table 1**
		(c) Summarise follow-up time (eg, average and total amount) **Page 5**
Outcome data	15*	Report numbers of outcome events or summary measures over time **Page 4**
Main results	16	(*a*) Give unadjusted estimates and, if applicable, confounder-adjusted estimates and their precision (eg, 95% confidence interval). Make clear which confounders were adjusted for and why they were included **Tables 2**
		(*b*) Report category boundaries when continuous variables were categorized **NA**
		(*c*) If relevant, consider translating estimates of relative risk into absolute risk for a meaningful time period **NA**
Other analyses	17	Report other analyses done—eg analyses of subgroups and interactions, and sensitivity analyses **page 8 and Supplementary Tables 1-6.**
**Discussion**		
Key results	18	Summarise key results with reference to study objectives **Pages 8 and 9**
Limitations	19	Discuss limitations of the study, taking into account sources of potential bias or imprecision. Discuss both direction and magnitude of any potential bias **Page 11**
Interpretation	20	Give a cautious overall interpretation of results considering objectives, limitations, multiplicity of analyses, results from similar studies, and other relevant evidence **Page 11**
Generalisability	21	Discuss the generalisability (external validity) of the study results **Page 12**
**Other information**		
Funding	22	Give the source of funding and the role of the funders for the present study and, if applicable, for the original study on which the present article is based **Page 13**

*Give information separately for exposed and unexposed groups.

Note: An Explanation and Elaboration article discusses each checklist item and gives methodological background and published examples of transparent reporting. The STROBE checklist is best used in conjunction with this article (freely available on the Web sites of PLoS Medicine at http://www.plosmedicine.org/, Annals of Internal Medicine at http://www.annals.org/, and Epidemiology at http://www.epidem.com/). Information on the STROBE Initiative is available at http://www.strobe-statement.org.

For peer review only

For peer review only - http://bmjopen.bmj.com/site/about/guidelines.xhtml
